# Integrin α7-Driven Senescence-Associated Follicular Helper T Cell Adhesion Induces Tertiary Lymphoid Structures in Nishiura Mice

**DOI:** 10.3390/ijms27167307

**Published:** 2026-08-16

**Authors:** Hiroshi Nishiura, Naoto Azuma, Kiyoshi Matsui, Teppei Hashimoto, Yoshiro Naito, Wenting Chen, Mai Imasaka, Masaki Ohmuraya

**Affiliations:** 1 Department of Genetics, Hyogo Medical University, 1-1, Mukogawa-cho, Nishinomiya, Hyogo 663-8501, Japan; we-chen@hyo-med.ac.jp (W.C.); ma-imasaka@hyo-med.ac.jp (M.I.); ohmuraya@hyo-med.ac.jp (M.O.); 2 Division of Diabetes, Endocrinology and Clinical Immunology, Department of Internal Medicine, Hyogo Medical University, 1-1, Mukogawa-cho, Nishinomiya, Hyogo 663-8501, Japan; naoazuuh@hyo-med.ac.jp (N.A.); k-matsui@hyo-med.ac.jp (K.M.); te-hashimoto@hyo-med.ac.jp (T.H.); 3 Department of Cardiovascular and Renal Medicine, Hyogo Medical University, 1-1, Mukogawa-cho, Nishinomiya, Hyogo 663-8501, Japan; ynaito@hyo-med.ac.jp

**Keywords:** integrin α7, laminin α4, Nishiura mice, Sjögren’s disease, senescence-associated follicular helper T cells, tertiary lymphoid structure

## Abstract

Senescence-associated follicular helper T (SA-Tfh) cells contribute to tertiary lymphoid structure (TLS) formation in autoimmune diseases, yet the mechanisms guiding their localization to glandular niches remain unclear. In the Nishiura (NI) Sjögren’s disease (SjD) mouse model, we demonstrated that mouse SA-Tfh (mSA-Tfh; CD4^+^/PD-1^+^/CD153^+^) cells form TLSs specifically within the submandibular gland niche. We identified integrin α7 (ITGα7) expression in mSA-Tfh cells and examined its functional role. Adhesion of mSA-Tfh cells to laminin (LAM) α2/α3/α4 was inhibited by anti-ITGα7 IgG (E-2), and ITGα7-deficient mSA-Tfh cells had reduced adhesion to LAMα1/α2 compared with LAMα3/α4/α5. Osteopontin production by mSA-Tfh cells, either alone or via interactions with mB220^+^/PD-1^+^ cells, was suppressed by E-2, anti-LAMα4 IgG, and ITGα7 deficiency. In female NI mice, E-2 administration at 24 weeks reduced Greenspan grade 4 TLSs at 28 weeks by decreasing ITGα7^+^/CD153^+^ cell accumulation. mSA-Tfh infiltration into aged C57BL/6 mice was similarly diminished by ITGα7 or LAMα4 deficiency. In human SjD cohorts, ITGα7 expression positively correlated with CD153 expression (R^2^ = 0.41) in peripheral lymphocytes and with LAMα4 expression (R^2^ = 0.35) in salivary gland vasculature and ducts. These findings suggest that circulating ITGα7^+^ SA-Tfh cells adhere to the LAMα4^+^ glandular niche to promote TLS formation in SjD.

## 1. Introduction

Tertiary lymphoid structures (TLSs), which increase with age, have been proposed as a mechanism that maintains immune functions after primary lymphoid organs such as the bone marrow and thymus undergo atrophy [1]. Similar to secondary lymphoid tissues, TLS follicles are formed through cell–cell interactions between B and T cells and generate memory T and B (mT/B) cells in response to antigenic stimulation by antigen-presenting cells [2,3]. However, the response of mT/B cells to specific autoantigens has not been elucidated fully.

We recently reported the Nishiura (NI) mouse model, which develops age-associated pathology resembling metabolic dysfunction–associated steatotic liver disease in males and Sjögren’s disease (SjD) in females [4,5]. Among mT/B cells, we identified mouse senescence-associated follicular helper T cells (mSA-Tfh; CD4^+^/PD-1^+^/CD153^+^) and mB220^+^/PD-1^+^ B cells (mSA-B). CD153 is a senescence-associated secretory phenotype (SASP) marker involved in the production of chronic inflammatory mediators. We demonstrated that mT/B cells form TLSs around CD31^+^ vascular endothelial cells in the proximal ducts of the submandibular gland [5]. Some TLSs also contained gp38^+^ lymphatic endothelial cells and integrin (ITG) α7^+^ pericytes, suggesting the presence of a vascular niche. However, little is known about vascular niches that support the maintenance and differentiation of mSA-Tfh and mSA-B cells.

Bone marrow niches provide distinct oxygen environments that shape stem cell behavior [6]. The vascular niche is hypoxic, where CXCL12-rich reticular cells and stem cell growth factor 1 maintain quiescence through hypoxia-inducible factors under low oxygen tension (10–20 mmHg). In contrast, the more oxygenated osteoblastic niche promotes stem cell activation and differentiation. Although these functional contrasts are recognized, the precise microarchitectural organization of each niche remains unclear [7,8].

The extracellular matrix (ECM) within vascular niches actively regulates cell fate. Fibronectin supports stem cell retention via ITGα5β1 binding to its RGD motif and mediates homing through ITGα4β1 recognition of the LDV motif. ITGαVβ3 on platelets contributes to angiogenesis through fibronectin adhesion [9,10]. Laminins (LAMs) further influence polarity and differentiation by engaging ITGα6β1, ITGα3β1, or dystroglycan [11]. Neural progenitors migrate through CXCL12/CXCR4 signaling and adhere to vascular laminins such as LAMα4β1γ1 or LAMα5β1γ1 [12], and LAMα5β1γ1–ITGα3β1 interactions suppress PTEN, promoting dopaminergic neuron survival [13]. How SA-immune cells use ECM cues to form TLSs remains largely unknown.

Recently, human hair follicle dermal papilla cells aggregate within a niche where they interact via ITGα7β1 with specific LAM isoforms containing α1, α2, or α4 chains and play a crucial role in hair follicle development and regeneration [14]. In addition, ITGα7 in primary monocyte-derived macrophages was shown to bind to LAMα2 and drive differentiation toward a dendritic cell-like phenotype [15]. In this study, we identified mouse ITGα7 (mITGα7) expression at the RNA level in mSA-Tfh cells in the spleens of 36-week-old female NI mice. We investigated the role of mITGα7 in mSA-Tfh-dependent TLS formation through interactions with mouse LAMs (mLAMs) in the salivary gland-specific microenvironment of female NI mice.

## 2. Results

### 2.1. mITGα7 Isoform 2 Expression in mSA-Tfh Cells

To investigate the roles of ITGs expressed on mSA-Tfh cells in TLS formation and SASP production through interactions with mSA-B cells in the submandibular glands, PD-1-negative and PD-1-positive CD4^+^ cells, CD8^+^ cells, F4/80^+^ cells, and B220^+^ cells were sorted from the spleens of 36-week-old female NI mice on a FACSAria III machine. mITG expression profiles were compared by RNA-based next-generation sequencing (NGS) analysis (Table 1). Several *ITGs* were expressed in PD-1-expressing immune cells. The strongest upregulation of *mITGα7* (16.22-fold) was observed in CD8^+^/PD-1^+^ cells. However, *mITGβ1* expression was not detected in this population. Conversely, a relatively high expression of *mITGβ1* (3.71-fold) was observed in CD4^+^/PD-1^+^ cells that reduced expression of mITGα subunits α1 and α6 was observed, whereas no such reduction was observed for α7. Therefore, this suggested the possibility of expressing mITGα7β1 in CD4^+^/PD-1^−^ and CD4^+^/PD-1^+^ cells.

**Figure 1:**
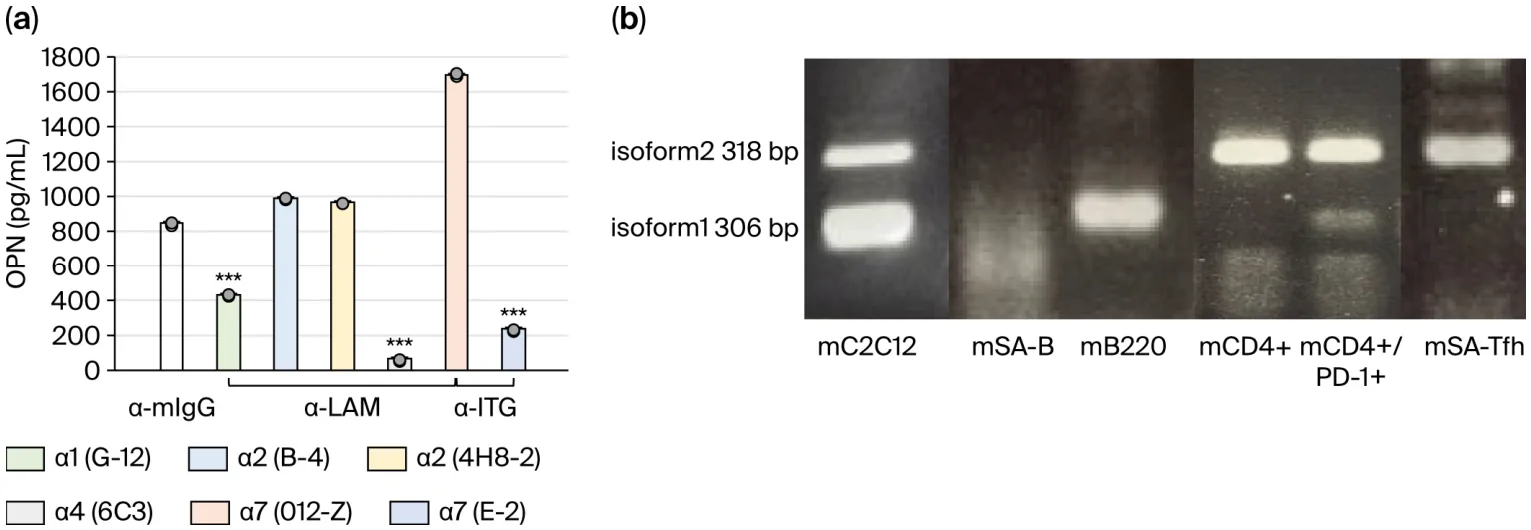
Expression of mITGα7 in mSA-Tfh Cells. (**a**) Mouse CD4^+^/PD-1^+^/CD153^+^ mSA-Tfh cells and B220^+^/PD-1^+^ mSA-B cells (2 × 10^4^ cells each) derived from the spleens of 36-week-old Nishiura (NI) mice were co-cultured for 48 h, and the OPN concentration in the culture supernatant was measured by sandwich ELISA. Circles represent individual data points: white, green, blue, yellow, red, and light blue columns indicate mean values from three independent experiments using anti-LAMα1 (G-12), anti-LAMα2 (B-4 and 4H8-2), anti-LAMα4 (6C3), and anti-ITGα7 (12-Z and E-2) mIgGs. (**b**) Control mC2C12 cells, mCD4^+^/PD-1^−^ (mCD4^+^), mCD4^+^/PD-1^+^/CD153^−^ (CD4^+^/PD-1^+^), mSA-Tfh, mB220^+^/PD-1^−^ (mB220^+^), and mSA-B cells were isolated from the spleens of 36-week-old NI mice. Genomic DNA from each population was used as a template for the PCR amplification of *mITGα7 isoforms* using S13-F and S13-R primers, generating a 306 bp product for *isoform 1* and a 318 bp product for *isoform 2*. PCR products were resolved on 2.5% agarose gels. Statistical significance was assessed using parametric tests (Tukey’s test) with multiple comparisons. *** *p* < 0.001.

To examine the role of mITGα7 in osteopontin (OPN) production by mSA-Tfh cells through interactions with mLAMs during co-culture with mSA-B cells, CD4^+^/PD-1^+^/CD153^+^ cells were isolated from the spleens of 36-week-old female NI mice and cocultured with B220^+^/PD-1^+^ cells for 48 h (Figure 1a). The concentration of OPN in the culture supernatant was 844 pg/mL. Anti-LAMα4 mIgG (6C3; −85.86%), anti-LAMα1 mIgG (G-12; −48.79%), and anti-ITGα7 mIgG (E-2; −46.07%) significantly suppressed OPN production in this order. In contrast, anti-ITGα7 mIgG (012-Z), anti-LAMα2 mIgG, and rat IgG (B-4 and 4H8-2) did not reduce OPN levels. To identify mITGα7 expression in mSA-Tfh and mSA-B cells, spleens from 36-week-old female NI mice were fractionated using a FACSAria III machine (Figure 1b). PCR analysis of RNA from these populations using S13 primers identified 306 bp *mITGα7 isoform 1* and 318 bp *mITGα7 isoform 2*. Expression of *mITGα7 isoform 2* was confirmed in mC2C12 cells, which are considered skeletal muscle precursor cells. The mC2C12 population exhibited *high mITGα7 isoform 1* and *low mITGα7 isoform 2* expression, suggesting the coexistence of mature and immature cells. In the spleen, *mITGα7 isoform 2* was highly expressed in CD4^+^/PD-1^−^ (mCD4^+^), CD4^+^/PD-1^+^/CD153^−^ (mCD4^+^/PD-1^+^), and CD4^+^/PD-1^+^/CD153^+^ (mSA-Tfh) cells. In contrast, *mITGα7 isoform 1* was expressed at low levels in mCD4^+^/PD-1^+^ cells and at high levels in B220^+^/PD-1^−^ (mB220^+^) cells. Notably, *mITGα7* expression was absent in B220^+^/PD-1^+^ (mSA-B) cells.

Collectively, these findings indicate that *mITGα7 isoform 2* is expressed in memory mSA-Tfh cells, as well as in mature physiological mCD4^+^ and aged mCD4^+^/PD-1^+^ cells.

### 2.2. Adhesion of mSA-Tfh Cells via mITGα7

**Figure 2:**
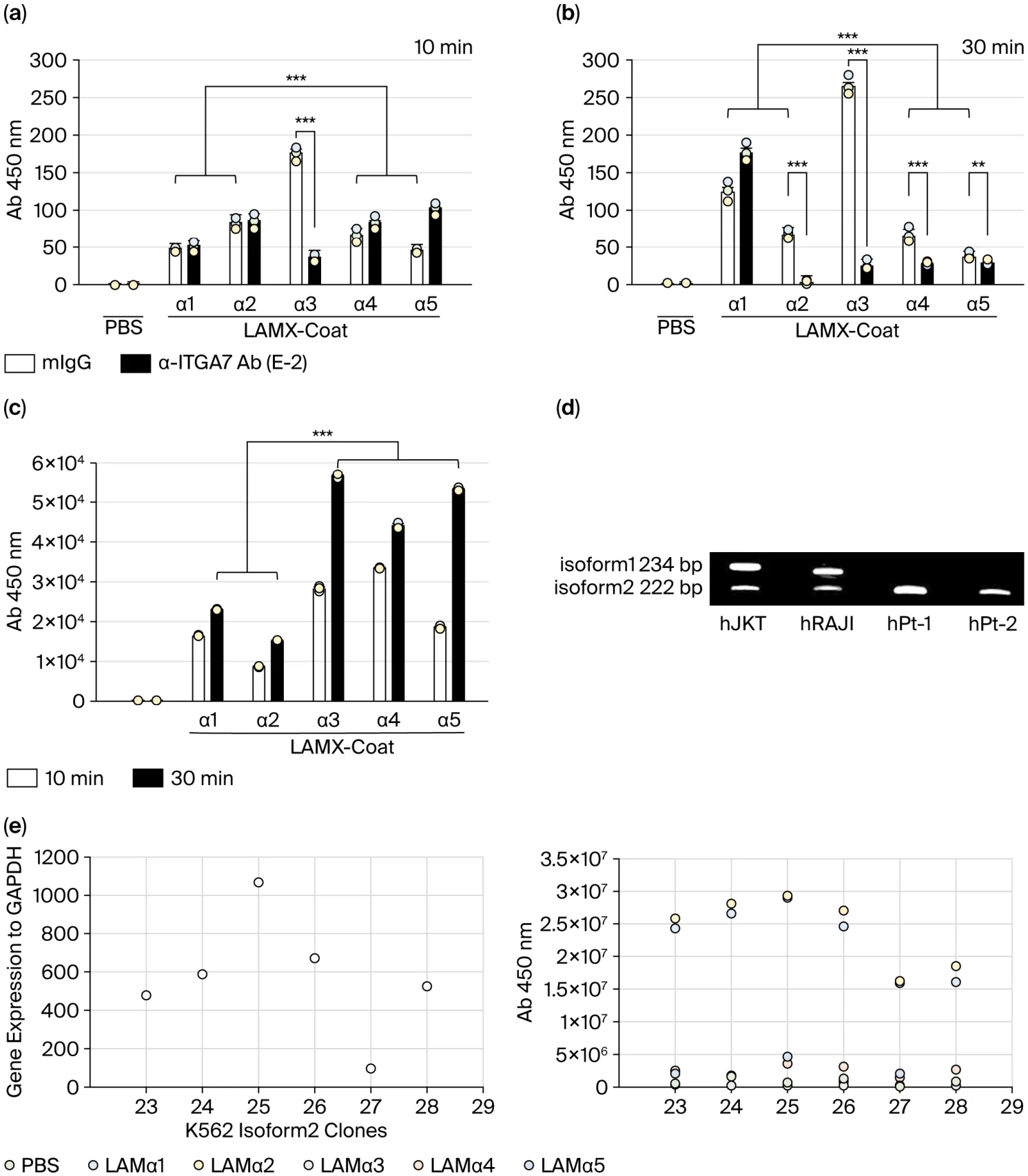
Adhesion of mSA-Tfh Cells Mediated by mITGα7. (**a**,**b**) Mouse mSA-Tfh cells (1 × 10^3^ cells) derived from 36-week-old NI mice were cultured for 20 min at 37 °C in flat-bottom 96-well plates pre-coated with PBS or various iMatrix proteins (0.155 μg/mL). After 10 or 30 min, the wells were washed once, and 100 μL of medium and 10 μL of WST-8 were added. The number of adherent cells was quantified by measuring absorbance at 450 nm. Circles indicate individual data points, and white and black bars represent the mean values from three experiments using mIgG or anti-ITGα7 mIgG (E2). (**c**) mSA-Tfh cells (1 × 10^3^ cells) were isolated from the spleens of 48-week-old ITGα7 KO mice. After 10 min (white bars) or 30 min (black bars), adherent cells were quantified by WST-8 at 450 nm. Circles represent individual data points: white and black columns indicate mean values from three independent experiments. (**d**) Genomic DNA from human JKT (hJKT) cells, hRAJI cells, and peripheral blood leukocytes from SjD patients was used for the PCR amplification of *hITGα7 isoforms* using S12-F and S12-R primers, generating 234 bp (*isoform 1*) and 222 bp (*isoform 2*) products. PCR products were resolved on 2.5% agarose gels. (**e**) hK562 cells (1 × 10^5^) were transduced with lentivirus (MOI 10), and clones with increased *hITGα7 isoform 2* expression relative to *GAPDH* were selected by qPCR. For adhesion assays, hK562^hITGα7 isoform2^ cells (2 × 10^4^) were seeded onto pre-coated plates. After 30 min, adherent cells were quantified by WST-8 at 450 nm. Circles represent predominant data points from three independent experiments. Statistical significance was assessed using parametric tests (Tukey’s test) with multiple comparisons. ** *p* < 0.01, *** *p* < 0.001.

To elucidate the mechanisms involved in the adhesion of mSA-Tfh cells to mLAMα4 mediated by mITGα7, mSA-Tfh cells were isolated from the spleens of female NI mice and pre-treated with either mIgG or anti-ITGα7 mIgG (E-2). Compared with adhesion to uncoated plates, the number of mSA-Tfh cells adhering to LAMα3 at 10 and 30 min was higher than adhesion to LAMα1, LAMα2, LAMα4, or LAMα5 (Figure 2). In a short-term assay, E-2 markedly suppressed adhesion to LAMα3 (−79.01%) but unexpectedly enhanced adhesion to LAMα5 (+42.04%) (Figure 2a). In a long-term assay, adhesion to LAMα2, LAMα4, and LAMα5 remained unchanged. E-2 suppressed adhesion to LAMα3 (−90.68%) as well as to LAMα2 (−95.5%) and LAMα4 (−58.88%), while promoting adhesion to LAMα1 (+42.04%) (Figure 2b). To validate these findings, mSA-Tfh^mITGα7−/−^ cells were isolated from the spleens of 48-week-old mITGα7 KO C57BL/6 mice (Figure 2c). Within 10 min, the adhesion of mSA-Tfh^mITGα7−/−^ cells to LAMα1, LAMα2, and LAMα5 was lower than that to LAMα3 and LAMα4. After 30 min, adhesion to LAMα1 and LAMα2 remained lower than that to LAMα3, LAMα4, and LAMα5.

To assess the homology between the *mITGα7 isoform 2* on mSA-Tfh cells and the *hITGα7 isoform 1* on hSA-Tfh cells, we performed PCR analysis using S12 primers and detected 234 bp *hITGα7 isoform 1* and 222 bp *hITGα7 isoform 2* (Figure 2d). In immature leukemia cell lines hJKT and hRAJI, which are different from memory hSA-Tfh cells, we detected *hITGα7 isoform 1^high^* and *hITGα7 isoform 2^low^*. In contrast, *hITGα7 isoform 2* was exclusively detected in peripheral blood leukocytes from two patients with SjD. These data suggest low numbers of circulating *hITGα7 isoform 1^+^* hSA-Tfh cells in SjD patients. To further evaluate the binding activity of hITGα7 isoform 2, but not isoform 1, hITGα7 isoform 2 was overexpressed in erythroid hK562 cells, which do not endogenously express hITGα7. Gene expression levels were quantified relative to *hGAPDH* across several clones (Figure 2e). In representative experiments, hITGα7 isoform 2^+^ hK562 cells adhered more strongly to LAMα1 and LAMα2 than to LAMα3, LAMα4, or LAMα5.

Collectively, these findings suggest that mouse and human ITGα7 expressed on SA-Tfh cells exhibit non-isoform-restricted and non-ligand-specific adhesion to LAMα1/α2/α3/α4, indicating a broader and more flexible ITG-LAM interaction than previously appreciated.

### 2.3. Downstream Signaling of mITGα7 in mC2C12 Cells

**Figure 3:**
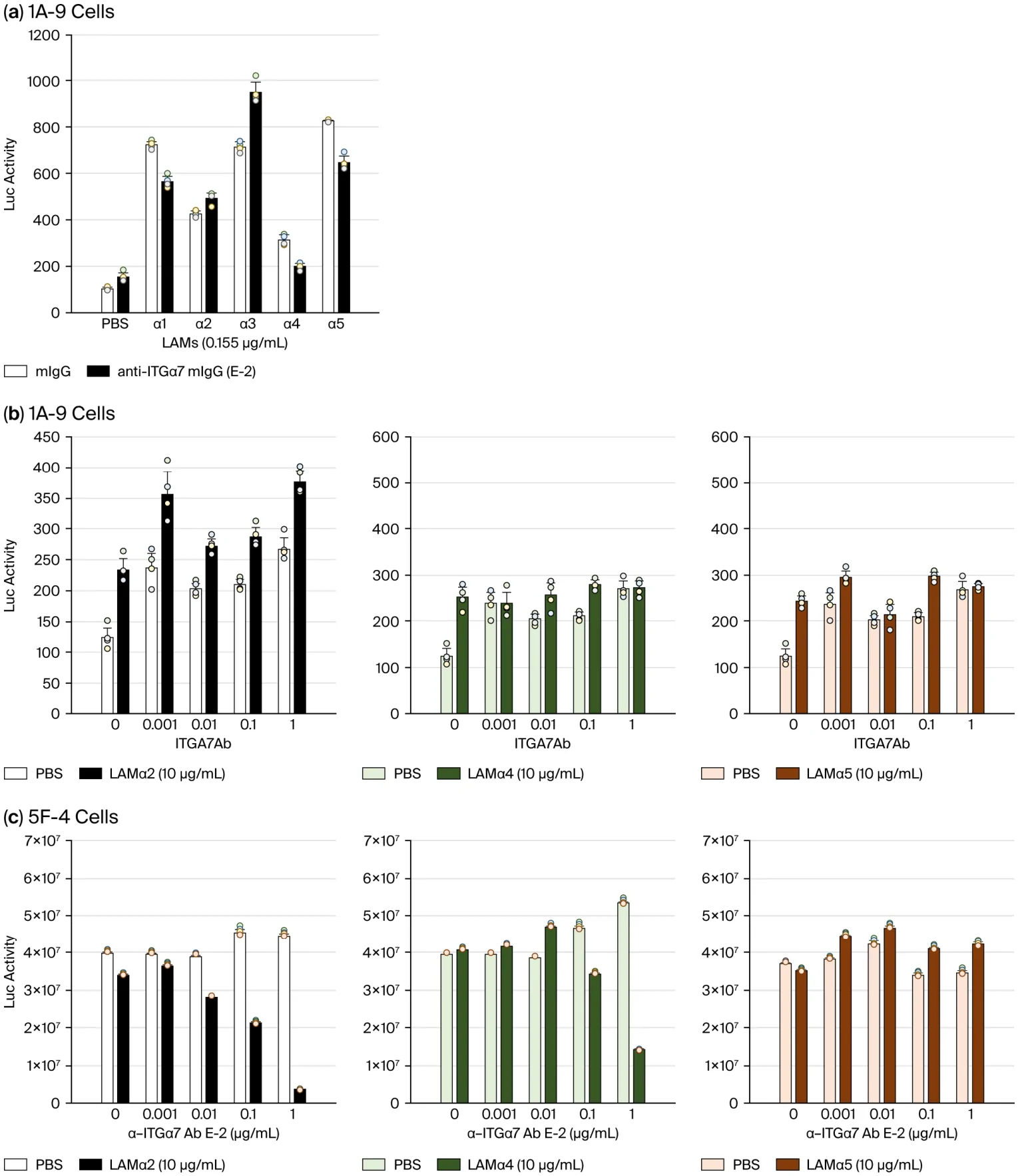
Downstream Signaling of mITGα7 in mC2C12 Cells. Mouse mC2C12 cells (1 × 10^5^) were seeded in 6-well plates, transfected with Lipofectamine and pGL4.33 or pGL4.34, and selected with Geneticin™. Stable clones (1A-9 and 5F-4) were chosen based on luciferase activity. (**a**) 1A-9 cells (2 × 10^4^) were cultured in 96-well flat-bottom plates pre-coated with PBS, iMatrix-111, iMatrix-221, iMatrix-332, iMatrix-411, or iMatrix-511 at 0.155 μg/mL at 37 °C in 5% CO_2_. After washing twice with PBS, 1A-9 cells with mIgG (white columns) or anti-ITGα7 mIgG (E-2; black columns) at 1 μg/mL were seeded. After 30 min, wells were washed once with PBS, and 100 μL medium containing 20% FBS was added. After 2 h, adhesion-dependent ERK activation was quantified as luciferase activity. (**b**,**c**) 1A-9 or 5F-4 cells (2 × 10^4^) were seeded in 96-well white plates and incubated for 24 h at 37 °C, 5% CO_2_. Cells were treated with 10 μL E-2 (0–1 μg/mL) for 30 min, followed by 10 μL iMatrix-221, -411, or -511 (10 μg/mL). After 2 h, 40 μL OneGlo medium was added, and luciferase activity for MAPK/ERK or RhoA activation was measured 2 h later using a GloMax 96 luminometer. Circles represent individual data points: columns indicate mean values from three independent experiments. Statistical significance was assessed using parametric tests (Tukey’s test) with multiple comparisons.

To investigate how mITGα7 on mSA-Tfh cells interacts with mLAMs to activate downstream signaling pathways, we generated 1A-9 and 5F-4 mC2C12 clones to assess MAPK/ERK and RhoA signaling. A total of 2 × 10^4^ 1A-9 cells, pre-treated with mIgG or anti-ITGα7 mIgG (E-2) at a final concentration of 1 μg/mL, were seeded onto plates coated with PBS, LAMα1, LAMα2, LAMα3, LAMα4, or LAMα5 (0.155 μg/mL) (Figure 3a). After 30 min, adhesion-dependent ERK activation was quantified by luciferase activity induced by 20% FBS. Pretreatment with E-2 partially inhibited adhesion to LAMα4 (−36%), LAMα1 (−21%), and LAMα5 (−19%), in this order. We next examined downstream signaling following stimulation with LAMα2, LAMα4, or LAMα5. A total of 2 × 10^4^ 1A-9 cells were seeded onto PBS-coated plates and stimulated with PBS or 10 μg/mL of LAMα2, LAMα4, or LAMα5 (Figure 3b). Luciferase activity induced by PBS was approximately 120, whereas stimulation with LAMα2, LAMα4, or LAMα5 increased the activity to approximately 250. Notably, LAMα2-induced luciferase activity was significantly enhanced in the presence of E-2. In contrast, LAMα4- and LAMα5-induced luciferase activity remained unchanged across all E-2 concentrations.

Conversely, when 2 × 10^4^ 5F-4 cells were seeded onto PBS-coated plates and stimulated with PBS or 10 μg/mL of LAMα2, LAMα4, or LAMα5 (Figure 3c), the baseline luciferase activity induced by PBS (4.0 × 10^7^) was not altered. However, LAMα2- and LAMα4-induced luciferase activity was reduced by E-2 in a dose-dependent manner (−91.79% and −73.23%, respectively). LAMα5-induced luciferase activity was unaffected by E-2 at all concentrations.

Taken together, these findings suggest that the allosteric inhibition of mITGα7 by mLAMα2 but not by mLAMα4 may suppress endogenous RhoA signaling, thereby enhancing downstream MAPK/ERK activation.

### 2.4. OPN Production Mediated by mITGα7 on mSA-Tfh Cells

**Figure 4:**
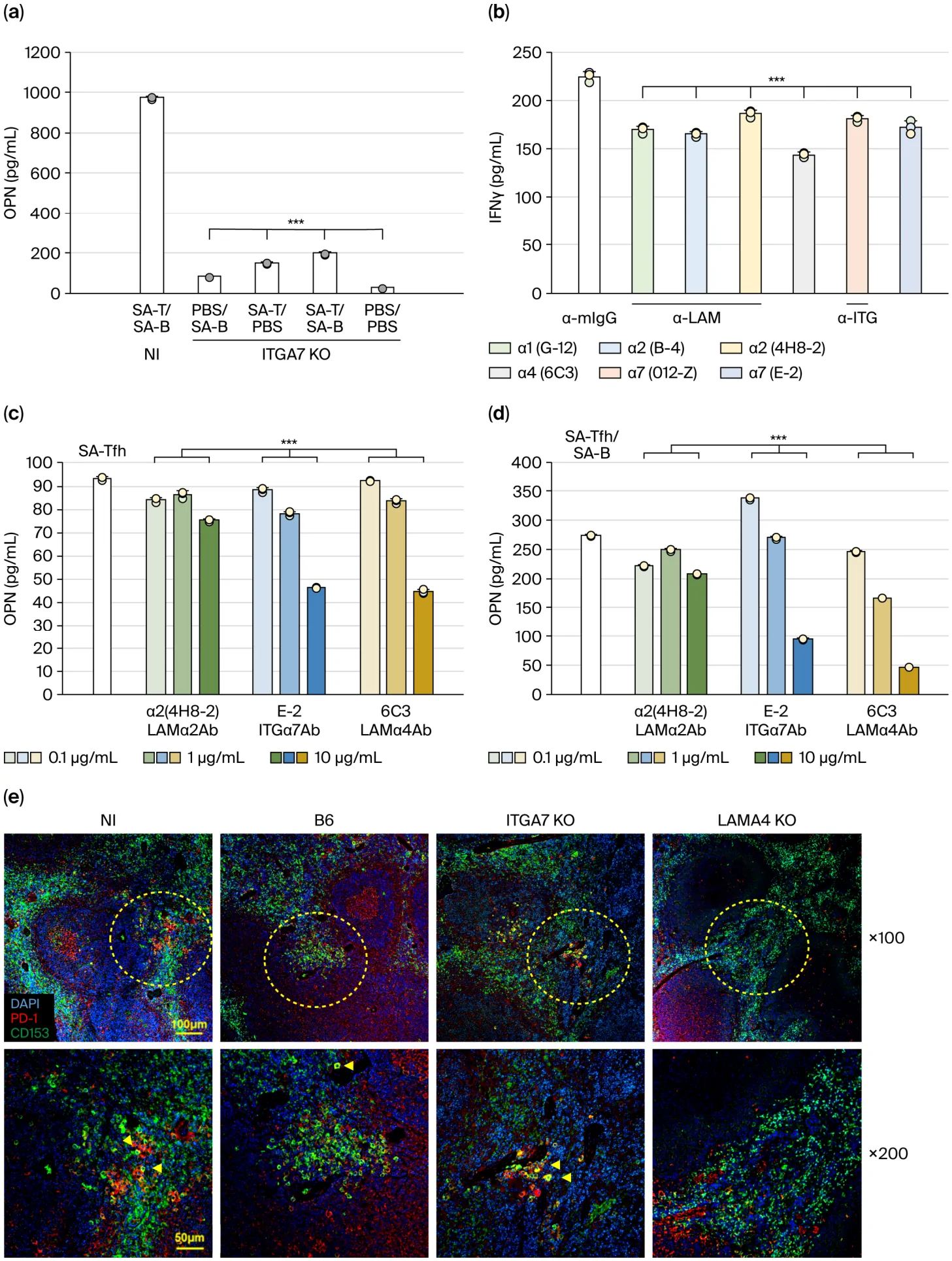
OPN Production Mediated by mITGα7 in mSA-Tfh Cells. (**a**) mSA-Tfh cells and mSA-B cells were isolated from the spleens of 36-week-old NI mice and 48-week-old ITGα7 KO mice. The following combinations were cultured in 96-well U-bottom plates (total 200 μL). After 48 h, OPN levels in the supernatants were quantified by sandwich ELISA. (**b**) mSA-Tfh and mSA-B cells (2 × 10^4^ each) isolated from 36-week-old NI mice were co-cultured in 96-well U-bottom plates. IFNγ levels were measured by sandwich ELISA. Circles represent individual data points: white, green, blue, yellow, red, and light blue columns indicate mean values from three independent experiments using mIgG, anti-LAMα1 (G-12), anti-LAMα2 (B-4 or 4H8-2), anti-LAMα4 (6C3), or anti-ITGα7 (012-Z or E-2) mIgGs at 1 μg/mL. (**c**,**d**) mSA-Tfh cells (2 × 10^4^) without or with mSA-B cells were cultured. After 48 h, OPN levels in the supernatants were quantified by sandwich ELISA. Circles represent individual data points: white, green, blue, yellow, red, and light blue columns indicate mean values from three independent experiments using mIgG (open column), 4H8-2 (small, middle and large green dotted columns), E-2 (small, middle and large blue dotted columns), or 6C3 (small, middle and large brown dotted columns) at concentrations ranging from 0.1 to 10 μg/mL. (**e**) Paraffin sections of spleen of 24-week-old NI mice, B6 mice, ITGα7 KO mice, and LAMα4 KO mice were incubated at 4 °C for 24 h with anti-PD-1 mIgG and anti-CD153 rabbit IgG, followed by incubation at 22 °C for 2 h with Alexa Fluor™ 647-conjugated anti-mouse IgG and Alexa Fluor™ 488-conjugated anti-rabbit IgG, along with DAPI. Images were acquired using a Zeiss LSM780 confocal microscope. The dotted yellow circle indicates the area observed at 200× magnification within the area observed at 100× magnification. The yellow arrowhead indicates PD-1^+^/CD153^+^ double-positive cells. Statistical significance was assessed using parametric tests (Tukey’s test) with multiple comparisons. *** *p* < 0.001.

To determine the role of mITGα7 on mSA-Tfh cells in OPN production via interactions with mLAMα2 and/or mLAMα4 on mSA-B cells, mSA-Tfh cells isolated from the spleens of 36-week-old female NI mice or 48-week-old female mITGα7 KO mice were cocultured with mSA-B cells from 36-week-old female NI mice for 48 h (Figure 4a). The concentration of OPN in the culture supernatant was approximately 974 pg/mL. OPN production was almost completely abolished in cocultures using mITGα7 KO-derived mSA-Tfh cells (−97.24%), indicating that OPN production is strongly dependent on mITGα7 in combination with mSA-B cells.

To assess the specificity of this response, IFNγ levels were measured in cocultures of mSA-Tfh and mSA-B cells from the same NI mice. The IFNγ concentration was approximately 224 pg/mL after 48 h (Figure 4b). Although antibodies against mLAMs and mITGα7 reduced IFNγ production, the inhibitory effects were minimal, suggesting that IFNγ production is largely independent of mITGα7-mLAM interactions. To determine whether mSA-Tfh or mSA-B cells expressed mLAMα2 and/or mLAMα4 during the 48 h culture period, mSA-Tfh and mSA-B cells were isolated from 36-week-old NI mice and cultured separately. OPN levels were 88.46 pg/mL when mSA-Tfh cells were cultured alone, and this was reduced to 46.44 pg/mL by E-2 (Figure 4c). Similarly, OPN levels of 92.45 pg/mL were reduced to 44.72 pg/mL by 6C3. In contrast, OPN levels of 84.21 pg/mL were only slightly reduced to 75.38 pg/mL by 4H8-2. Thus, mITGα7-dependent and mLAMα4-dependent OPN production was estimated to be 42.02 pg/mL (88.46 − 46.44) and 47.73 pg/mL (92.45 − 44.72), respectively.

When mSA-Tfh cells were cocultured with mSA-B cells, OPN levels increased to 338.28 pg/mL and were reduced to 95.51 pg/mL by E-2 (Figure 4d). OPN levels of 246.66 pg/mL were reduced to 47.31 pg/mL by 6C3, whereas OPN levels of 221.92 pg/mL were only slightly reduced to 207.77 pg/mL by 4H8-2. The mITGα7-dependent and mLAMα4-dependent OPN production was therefore estimated to be 242.77 pg/mL (338.28 − 95.51) and 199.35 pg/mL (246.66 − 47.31), respectively. The similar inhibitory effects of E-2 and 6C3 suggest that mSA-Tfh and mSA-B cells express mLAMα4 and that mITGα7-mLAMα4 interactions contributed to OPN production during the 48 h co-culture. The mITGα7-dependent OPN production enhanced by mSA-B cells was estimated to be 151.62 pg/mL (199.35 − 47.73).

In 24-week-old female NI mice, PD-1^+^ cells were observed in the T cell and follicular regions (Figure 4e). Furthermore, a small number of PD-1^+^/CD153^+^ cells were found in the T cell and follicular boundary regions. In contrast to female NI mice, a small number of PD-1^+^/CD153^+^ cells were observed in the boundary region in 24-week-old female B6 mice. Conversely, the infiltration of PD-1^+^ and PD-1^+^/CD153^+^ cells was suppressed in ITGα7 and LAMα4 KO mice.

Collectively, these findings indicate that mITGα7 expressed on mSA-Tfh cells promote OPN production in the spleens of female NI mice.

### 2.5. In Vivo Effects of ITGα7 Deficiency on SA-Immune Cells in Female NI Mice

To investigate the role of mITGα7 in the maintenance of the mSA-immune cell system, bone marrow, thymus, spleen, and peripheral blood were collected from 15-week-old male and female B6 and mITGα7 KO mice.

**Figure 5:**
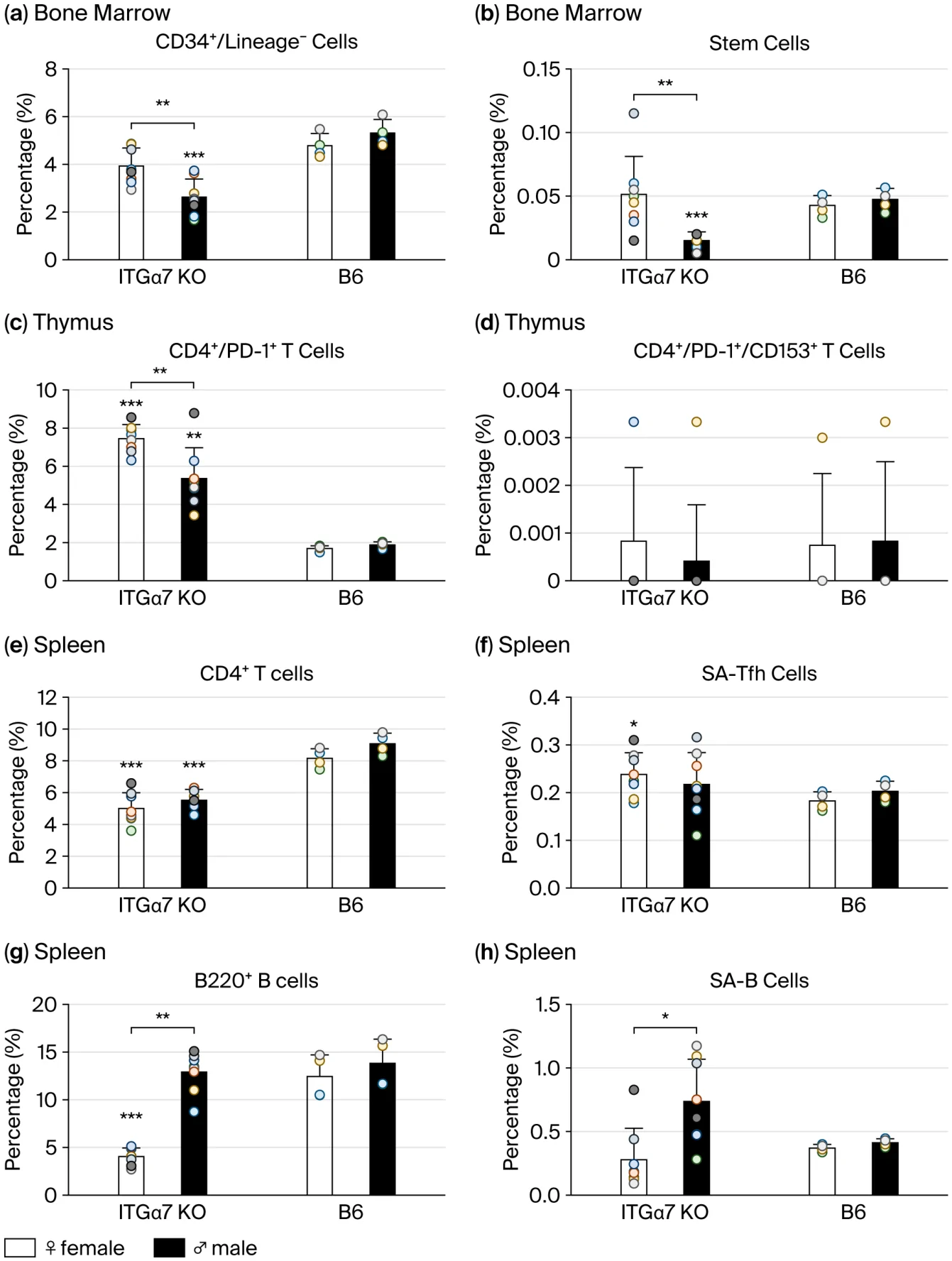

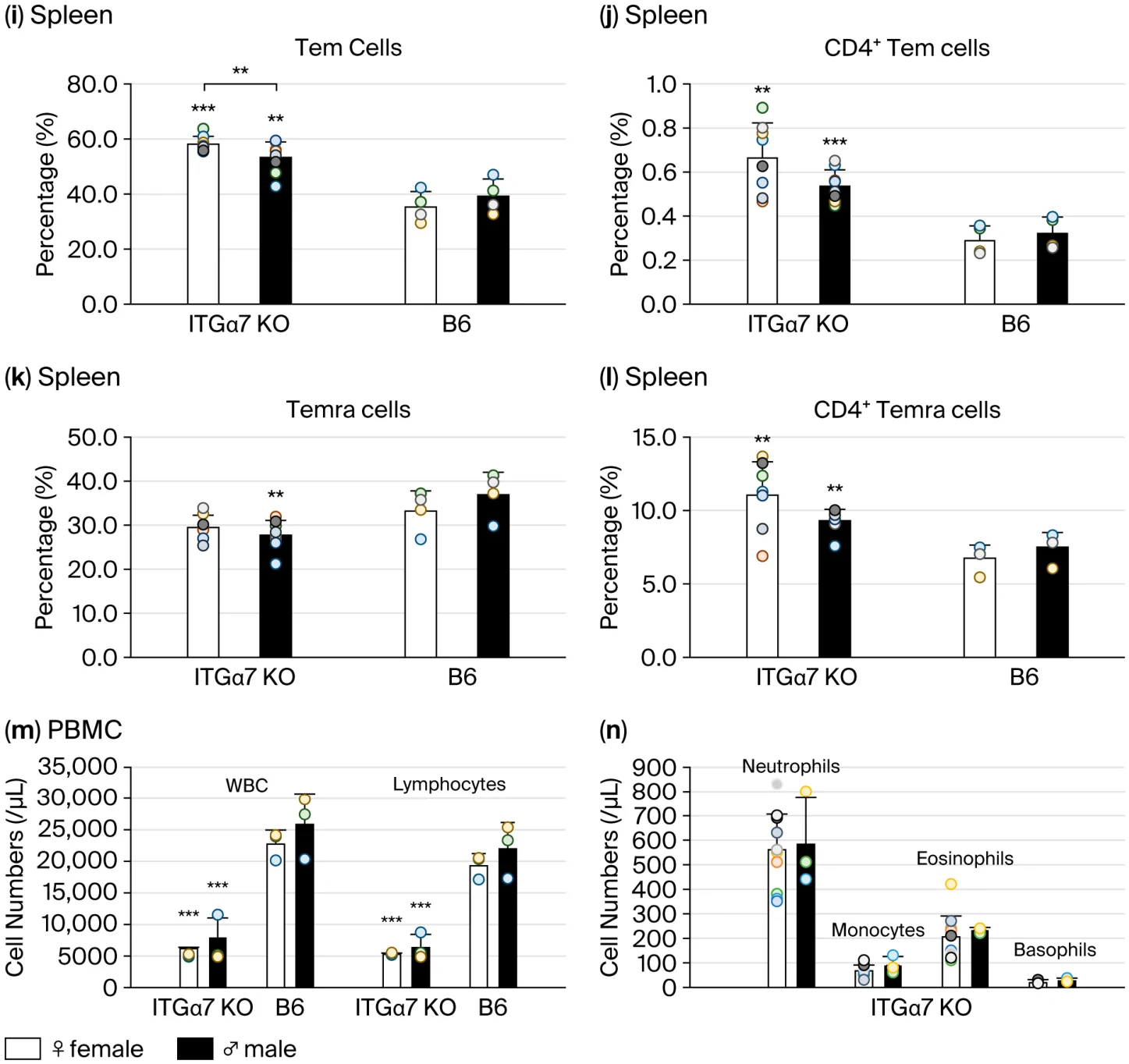
In Vivo Effects of ITGα7 Deficiency on SA-Immune Cells. The bone marrow, thymus, spleen, and peripheral blood were collected from 15-week-old C57BL/6 (B6) and ITGα7 KO female (white columns) and male (black columns) mice. (**a**) Lineage^−^/CD34^+^ cells and (**b**) lineage^−^/CD34^+^/c-Kit^+^/Sca-1^+^ stem cells were analyzed using a FACSAria III machine. (**c**) CD4^+^/PD-1^+^ cells and (**d**) CD4^+^/PD-1^+^/CD153^+^ cells were analyzed. (**e**) CD4^+^ cells, (**f**) CD4^+^/PD-1^+^/CD153^+^ cells, (**g**) B220^+^ cells, and (**h**) B220^+^/PD-1^+^ cells were analyzed. For memory T-cell analysis, splenic cells were stained. (**i**) CCR7^−^/CD45RA^−^ effector memory (Tem) cells, (**j**) CD4^+^ Tem cells, (**k**) CCR7^−^/CD45RA^+^ Temra cells, and (**l**) CD4^+^ Temra cells were analyzed. (**m**,**n**) Peripheral blood leukocytes and their subsets (neutrophils, monocytes, eosinophils, basophils) were quantified. Circles represent individual data points: columns indicate mean values from three independent experiments. Statistical significance was assessed using non-parametric tests (Wilcoxon test) with multiple comparisons. * *p* < 0.05, ** *p* < 0.01, *** *p* < 0.001.

In the microenvironment of the bone marrow, which is a primary lymphoid structure (PLS), no sex-dependent differences were observed in the proportion of CD34^+^/lineage^−^ cells in B6 mice (Figure 5a). In contrast, male mITGα7 KO mice exhibited a significant reduction compared with female mITGα7 KO mice, resulting in a markedly lower proportion of CD34^+^/lineage^−^ cells in male mITGα7 KO mice than in male B6 mice. Similarly, no sex-dependent differences were observed in the proportion of CD34^+^/lineage^−^/c-Kit^+^/Sca-1^+^ stem cells in B6 mice (Figure 5b). However, male mITGα7 KO mice showed a significant decrease compared with female mITGα7 KO mice, and the proportion of stem cells in male mITGα7 KO mice was significantly lower than in male B6 mice.

In the microenvironment of the other PLS, the thymus, the proportion of CD4^+^/PD-1^+^ T cells did not differ between males and females in the mITGα7 KO or B6 genotypes (Figure 5c). However, female and male mITGα7 KO mice exhibited significantly higher proportions of CD4^+^/PD-1^+^ T cells compared with male B6 mice. The proportion of CD4^+^/PD-1^+^/CD153^+^ T cells did not differ between the sexes of either genotype (Figure 5d), and no significant differences were observed between mITGα7 KO and B6 mice.

In the microenvironment of the spleen, a secondary lymphoid structure (SLS), the proportion of CD4^+^ T cells did not differ between sexes in the mITGα7 KO or B6 genotypes (Figure 5e). However, female and male mITGα7 KO mice had significantly reduced proportions compared with male B6 mice. The proportion of CD4^+^/PD-1^+^/CD153^+^ (SA-Tfh) cells did not differ between sexes of either genotype (Figure 5f), although female mITGα7 KO mice exhibited a significant increase compared with female B6 mice. The proportion of B220^+^ B cells showed no sex-dependent differences in B6 mice (Figure 5g). In mITGα7 KO mice, however, female mice exhibited significantly lower proportions than male mITGα7 KO mice and male B6 mice. Similarly, the proportion of B220^+^/PD-1^+^ (SA-B) cells showed no sex-dependent differences in B6 mice (Figure 5h), whereas mITGα7 KO male mice exhibited significantly higher proportions than female mITGα7 KO mice and male B6 mice. In B6 mice, the proportion of Ccr7^−^/CD45RA^−^ effector memory (Tem) cells did not differ between sexes (Figure 5i). In contrast, female mITGα7 KO mice exhibited significantly higher proportions than male mITGα7 KO mice, and male and female mITGα7 KO mice showed significantly increased Tem proportions compared with their B6 counterparts. For CD4^+^ Tem cells, no sex-dependent differences were observed in either genotype (Figure 5j), but male and female mITGα7 KO mice had significantly higher proportions than the B6 mice. The proportion of Ccr7^−^/CD45RA^+^ Temra cells did not differ between the sexes in B6 mice (Figure 5k). However, male mITGα7 KO mice exhibited significantly lower proportions than female mITGα7 KO mice and male B6 mice. For CD4^+^ Temra cells, no sex-dependent differences were observed in either genotype (Figure 5l), but male and female mITGα7 KO mice had significantly increased proportions compared with their respective B6 controls.

In the environment of peripheral blood circulating in the lymphatic system, total white blood cell (WBC) and lymphocyte counts did not differ between the sexes of either genotype (Figure 5m). However, female and male mITGα7 KO mice exhibited significantly reduced counts compared with B6 mice. Similarly, WBC subsets, including neutrophils, monocytes, eosinophils, and basophils, showed no sex-dependent differences in mITGα7 KO mice (Figure 5n).

Collectively, these findings suggest that the physiological immune cell maintenance system begins to shift toward an SA-immune cell-dominant state in mITGα7 KO mice as early as 15 weeks of age, indicating a critical role for mITGα7 in maintaining immune homeostasis.

### 2.6. In Vivo Effects of Anti-ITGα7 mIgG on SA-Dependent TLS Formation in Female NI Mice

**Figure 6:**
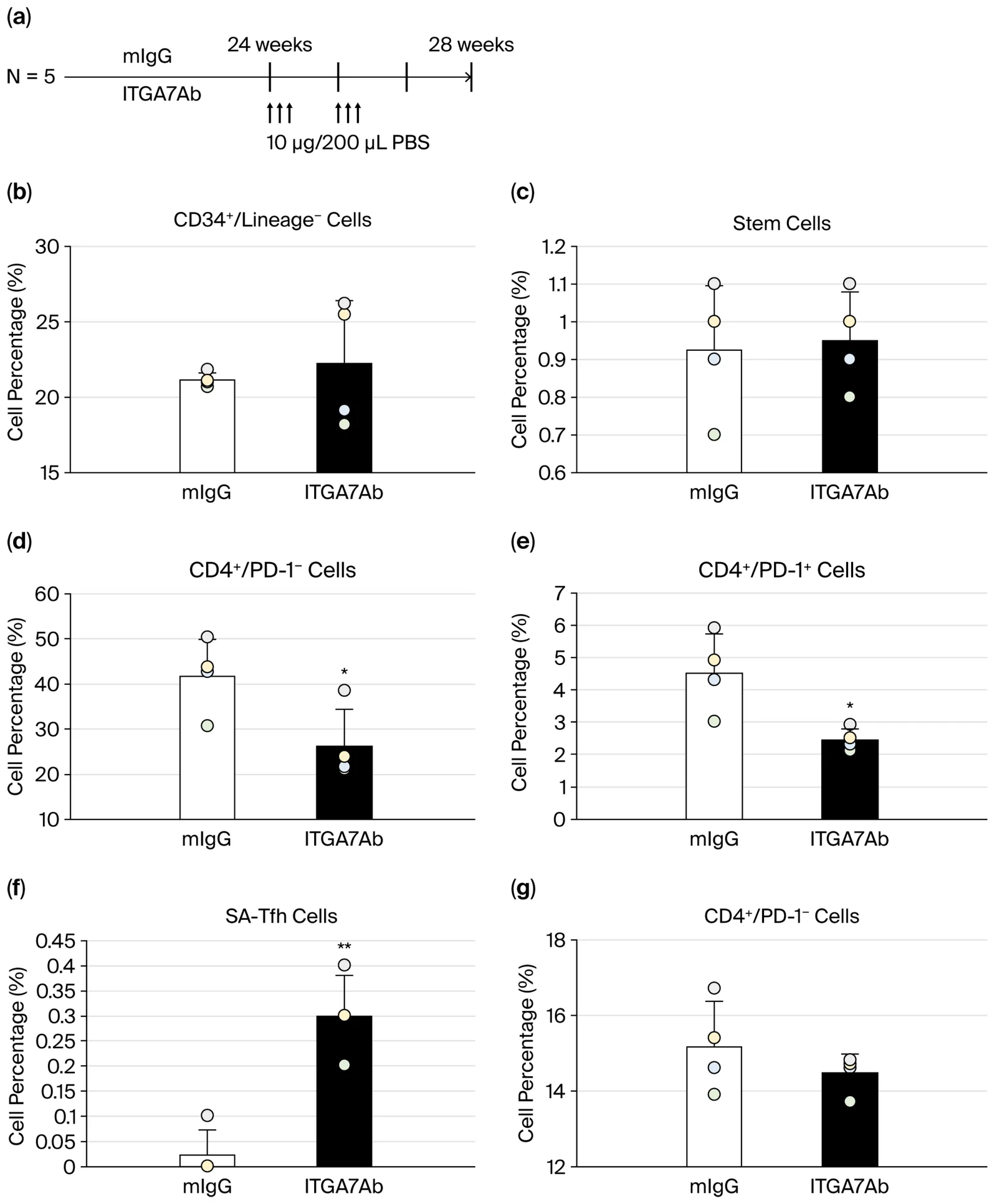

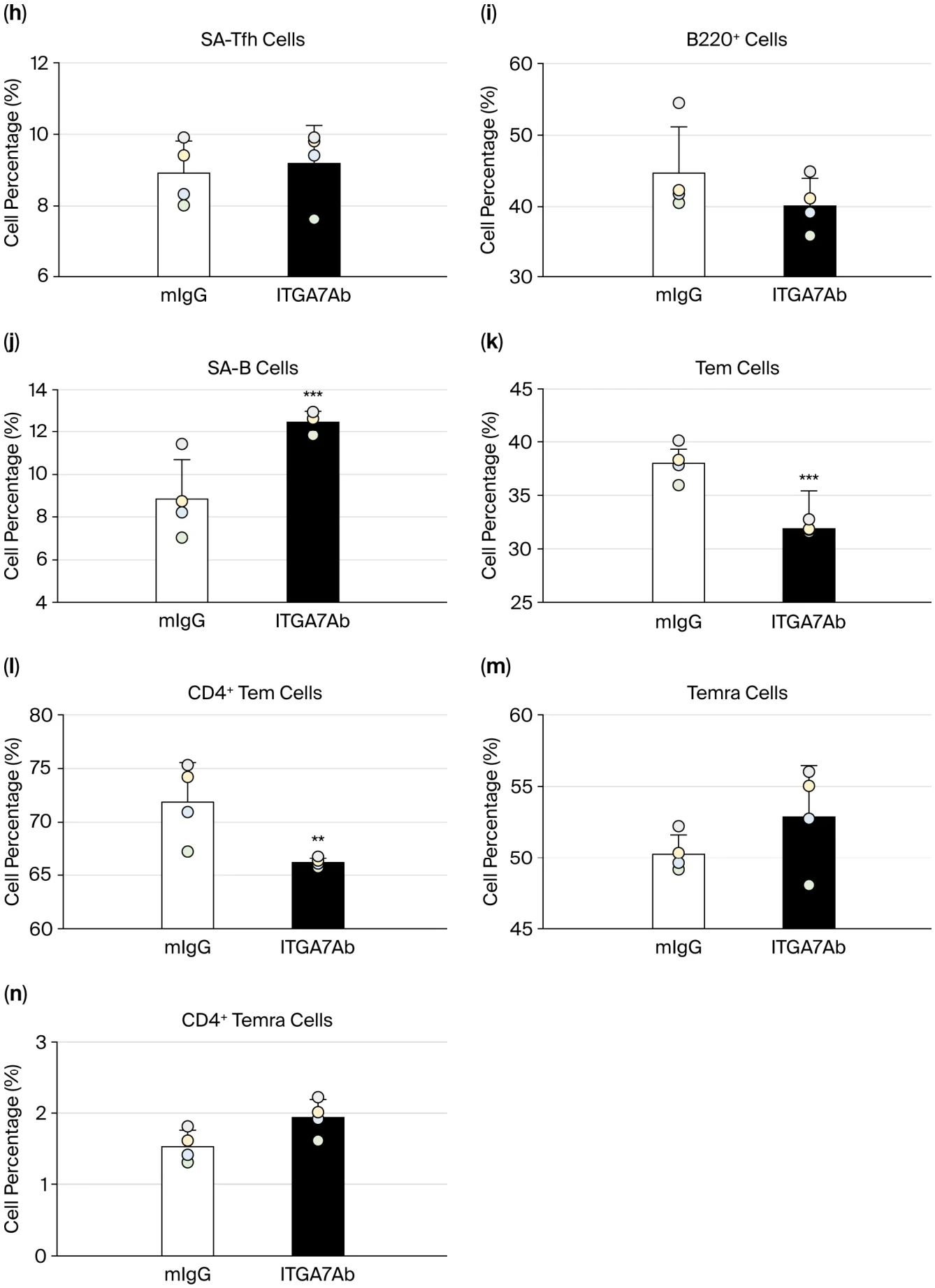

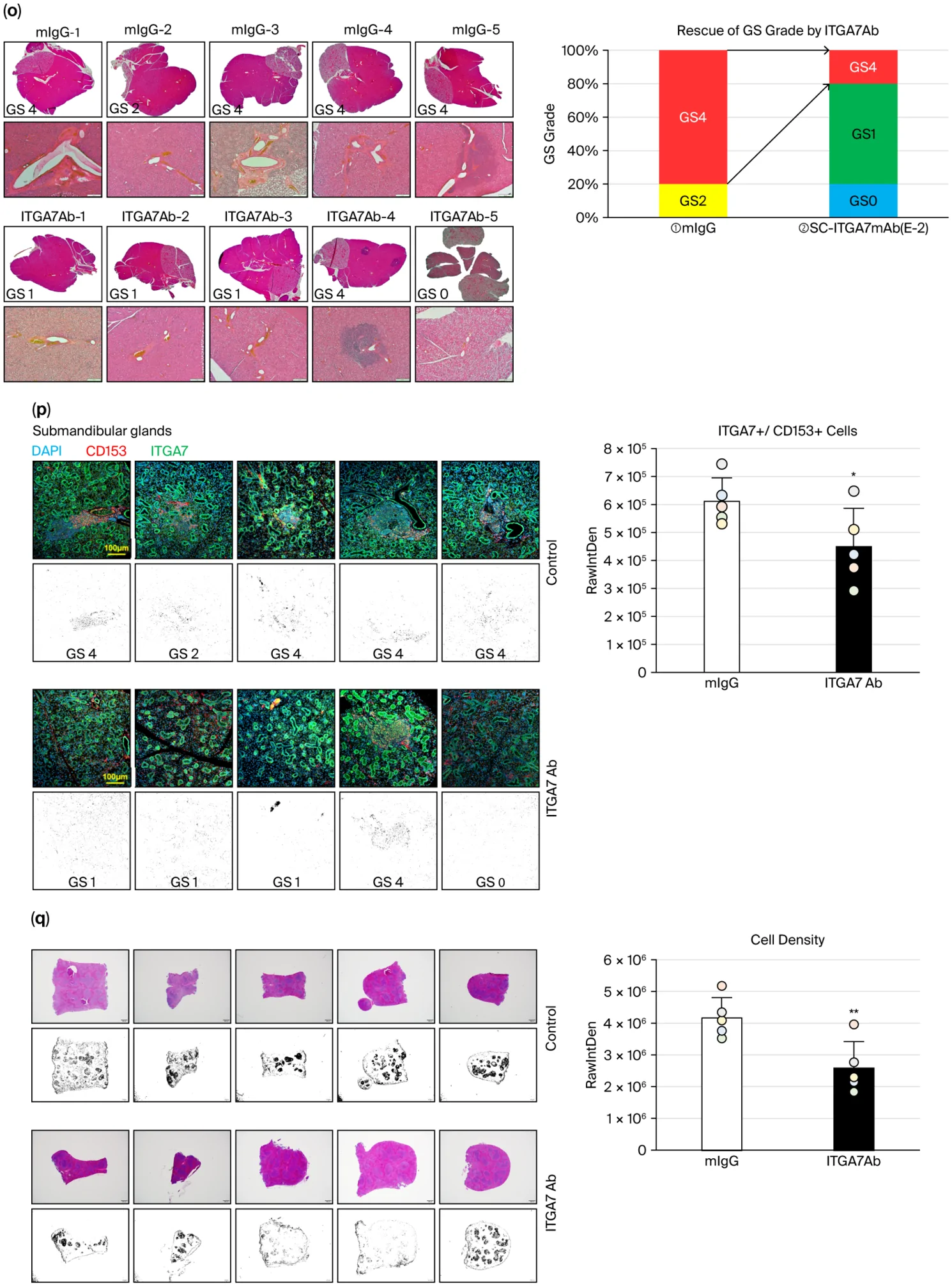

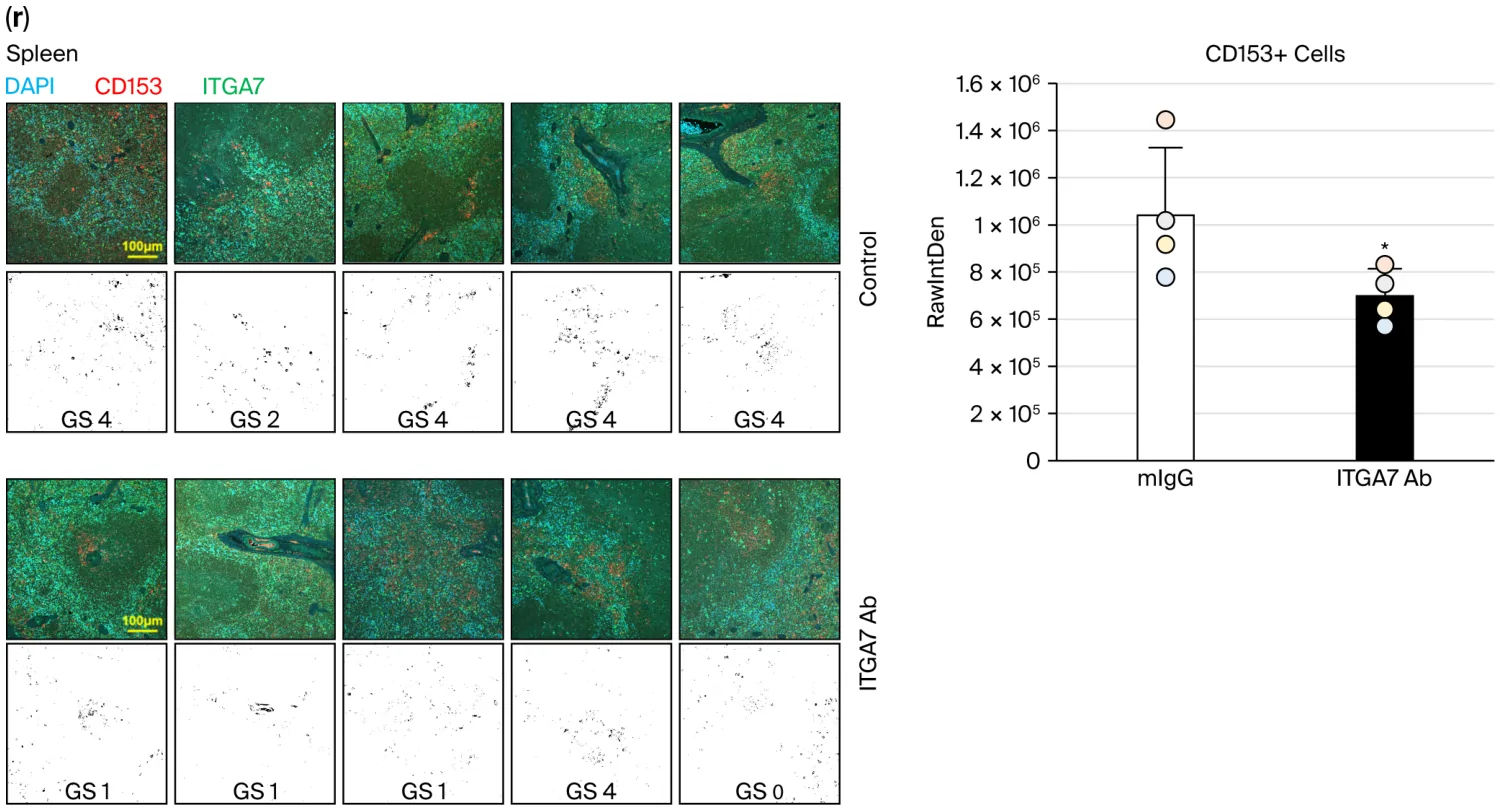
In Vivo Effects of Anti-ITGα7 mIgG on TLS Formation in Nishiura Female Mice. (**a**) NI mice were maintained on a standard diet until 24 weeks of age. Mouse IgG (mIgG; n = 5) or anti-ITGα7 mIgG (E-2; 10 μg/200 μL; n = 5) was administered intraperitoneally for 3 consecutive days at 24 and 25 weeks. The bone marrow, thymus, spleen, and submandibular glands were collected at 28 weeks. (**b**) Lineage^−^/CD34^+^ cells and (**c**) lineage^−^/CD34^+^/c-Kit^+^/Sca-1^+^ stem cells were analyzed using a FACSAria III machine. (**d**) CD4^+^/PD-1^−^ cells, (**e**) CD4^+^/PD-1^+^ cells, and (**f**) CD4^+^/PD-1^+^/CD153^+^ cells were analyzed. (**g**) CD4^+^/PD-1^+^ cells, (**h**) CD4^+^/PD-1^+^/CD153^+^ cells, (**i**) B220^+^/PD-1^−^ cells, and (**j**) B220^+^/PD-1^+^ cells were analyzed. (**k**) CCR7^−^/CD45RA^−^ effector memory (Tem) cells, (**l**) CD4^+^ Tem cells, (**m**) CCR7^−^/CD45RA^+^ Temra cells, and (**n**) CD4^+^ Temra cells were analyzed. (**o**) Submandibular glands were stained with H&E. Images were acquired using an Olympus BX53 microscope and cellSens software. TLS formation and cellular infiltration were evaluated using the GS classification. (**p**) Paraffin sections were incubated with anti-ITGα7 mIgG (E-2) and anti-CD153 rabbit IgG, followed by incubation at 22 °C for 2 h with Alexa Fluor™ 488-conjugated anti-mouse IgG and Alexa Fluor™ 647-conjugated anti-rabbit IgG, along with DAPI. Images were acquired using a Zeiss LSM780 confocal microscope. Double-positive cells were quantified using NIH ImageJ 64-bit Java 8. (RawIntDen). (**q**) Spleen sections were stained with H&E, imaged using BX53 microscope, and the cell density was quantified using NIH ImageJ (RawIntDen). (**r**) Adjacent spleen sections were stained with anti-ITGα7 mIgG (E-2) and anti-CD153 rabbit IgG, followed by Alexa Fluor™ 488/647 secondary antibodies and DAPI. Circles represent individual data points: columns indicate mean values from five independent experiments using mIgG (white columns) and anti-ITGα7 mIgG (E-2; black columns). Statistical significance was assessed using non-parametric tests (Wilcoxon test) with multiple comparisons. * *p* < 0.05, ** *p* < 0.01, *** *p* < 0.001.

To evaluate the involvement of mITGα7 in SA-dependent TLS formation in the submandibular glands of female NI mice (n = 10), the bone marrow, thymus, spleen, and submandibular glands were collected 4 weeks after the intraperitoneal administration of 10 μg mIgG or anti-ITGα7 mIgG (E-2) dissolved in 200 μL PBS for 3 consecutive days at 24 and 25 weeks of age (Figure 6a).

In 28-week-old female NI mice, the proportions of CD34^+^/lineage^−^ cells and CD34^+^/lineage^−^/Sca-1^+^/c-Kit^+^ stem cells in the bone marrow were not significantly altered by E-2 compared with mIgG (Figure 6b,c). In the thymus, CD4^+^/PD-1^−^ and CD4^+^/PD-1^+^ cell populations were reduced by E-2 compared with mIgG (Figure 6d,e). In contrast, the proportion of SA-Tfh cells increased following E-2 treatment (Figure 6f). In the spleen, CD4^+^/PD-1^+^ and SA-Tfh cell proportions were not significantly affected by E-2 (Figure 6g,h). Similarly, the proportion of B220^+^/PD-1^−^ B cells was unchanged (Figure 6i), whereas SA-B cells increased following E-2 administration (Figure 6j). Furthermore, Tem and CD4^+^ Tem populations were significantly reduced by E-2 (Figure 6k,l), whereas Temra and CD4^+^ Temra populations remained unchanged (Figure 6m,n). These findings suggest that E-2 may contribute to age-associated immune remodeling by reducing physiological CD4^+^ T cells and increasing mT cell and mB cell subsets.

In 28-week-old female NI mice treated with mIgG, TLSs consisting of more than 50 cells were frequently observed around the submandibular gland ducts, with GS4 lesions accounting for 80% and GS2 lesions (under 50 cells) accounting for 20% (Figure 6o). In contrast, E-2 treatment markedly reduced TLS formation: GS4 lesions decreased to 20%, GS1 lesions (mild infiltration without TLS) increased to 60%, and GS0 lesions (no infiltration) accounted for 20%. In mIgG-treated mice, mITGα7^+^, CD153^+^, and double-positive cells were moderately present within the TLSs (Figure 6p). E-2 treatment reduced the adhesion of mITGα7^+^, CD153^+^, and double-positive cells to the vasculature and diminished their infiltration into the glandular parenchyma. To investigate the role of ITGα7 in mCD153^+^ cells that infiltrate into the submandibular gland to form TLSs, and mCD153^+^ cells that form SLSs reconstructed in response to the physiological decrease in lymphocytes associated with aging, spleen sections were examined by H&E and immunofluorescence staining. The overall cell density of the spleen sections was decreased by E-2 administration (Figure 6q). However, the number of mITGα7^+^/CD153^+^ cells in the spleen T-cell region was also decreased after E-2 administration (Figure 6r).

Collectively, these findings indicate that mITGα7 functions as an adhesion molecule for physiological and SA-immune cells, contributing to immune maintenance in aging and promoting SA-dependent TLS formation in the submandibular glands of female NI mice.

### 2.7. Side Effects Associated with ITGα7 Deficiency

To confirm the absence of antibody-dependent cellular cytotoxicity (ADCC) activity mediated by anti-ITGα7 mIgG (E-2), cytotoxicity assays were performed using hRaji cells and mSA-Tfh cells expressing ITGα7 (Supplementary Figure S1). When hRaji cells were used as effector cells and CD20-expressing B cells as targets, E-2 exhibited no ADCC activity across the negative and positive control ranges, defined by mIgG and anti-CD20 antibodies. Similarly, when mSA-Tfh cells were used as effector cells, E-2 did not induce ADCC activity under the same conditions.

To evaluate potential physiological side effects associated with ITGα7 deficiency, electrocardiography and echocardiography were performed in mITGα7 KO mice at 12 weeks (young) and 42 weeks (aged) (Supplementary Figure S2). No significant differences in cardiac function were observed between the young and aged WT mice. In mITGα7 KO mice, aged animals exhibited a slight increase in left ventricular end-systolic diameter and a modest decrease in left ventricular fractional shortening compared with young mITGα7 KO mice. However, these changes did not significantly impair overall cardiac performance or blood pressure regulation.

Although the findings in this study were derived primarily from cells and two mice, it was predicted that ITGA7-targeted antibody therapy would not induce clinically serious cytotoxicity or cardiac dysfunction mediated by ADCC.

### 2.8. Cohort Study

To investigate whether hITGα7 might be a therapeutic marker for autoimmune diseases, peripheral blood samples were collected from outpatients at the Division of Diabetes, Endocrinology and Clinical Immunology, Department of Internal Medicine, Hyogo Medical University, and leukocyte fractions were isolated (Table 2). For each patient, the expression levels of *hPD-1*, *hCD153*, *hLAMα4*, and *hITGα7* were normalized to *hGAPDH* and expressed as ratios per lymphocyte. These values were analyzed separately for patients receiving medication and those who were not. Correlations were examined between *hPD-1*/Lymph and *hCD153*/Lymph, and between *hPD-1*/Lymph or *hCD153*/Lymph and *hITGα7*/Lymph or *hLAMα4*/Lymph.

**Figure 7:**
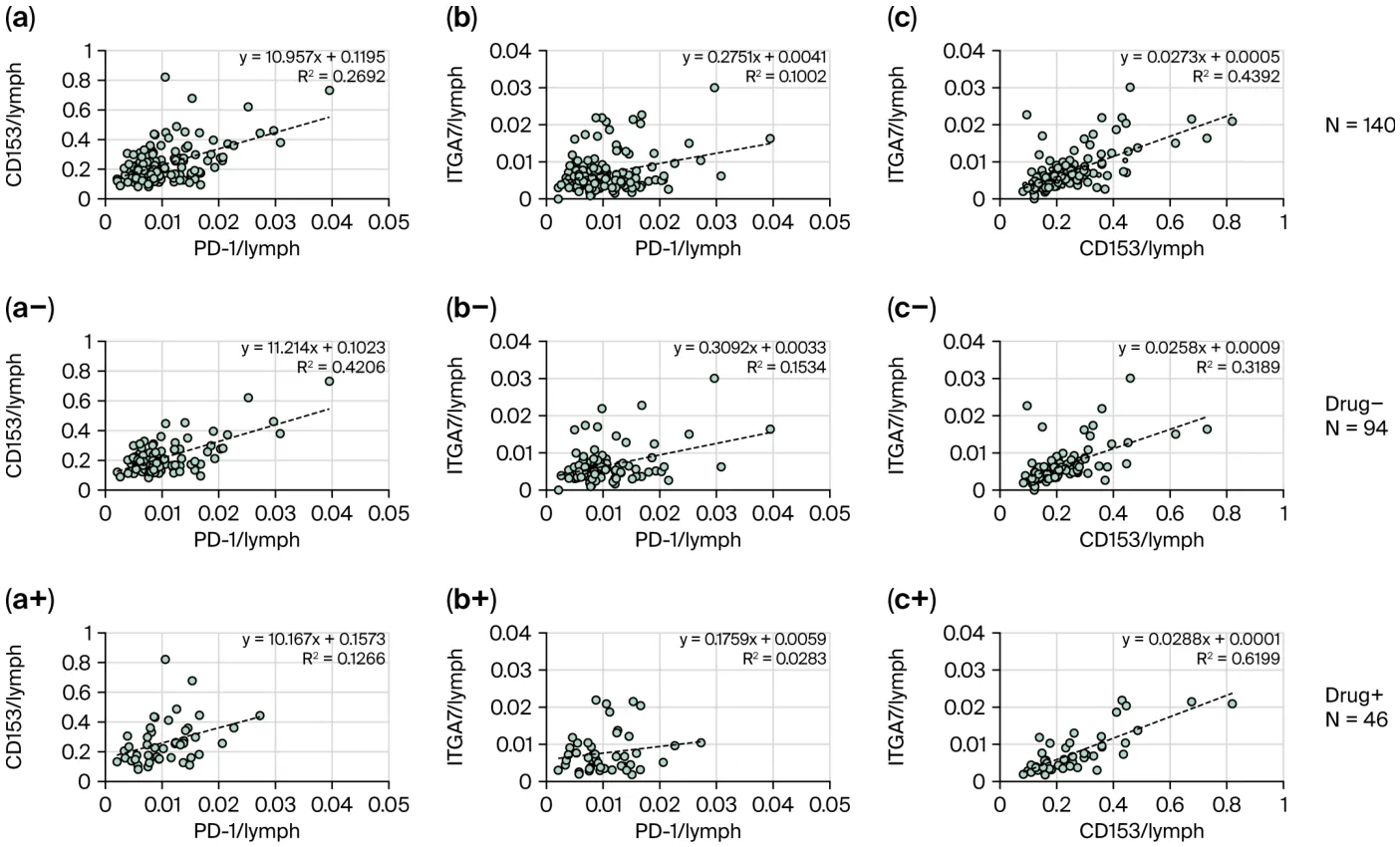

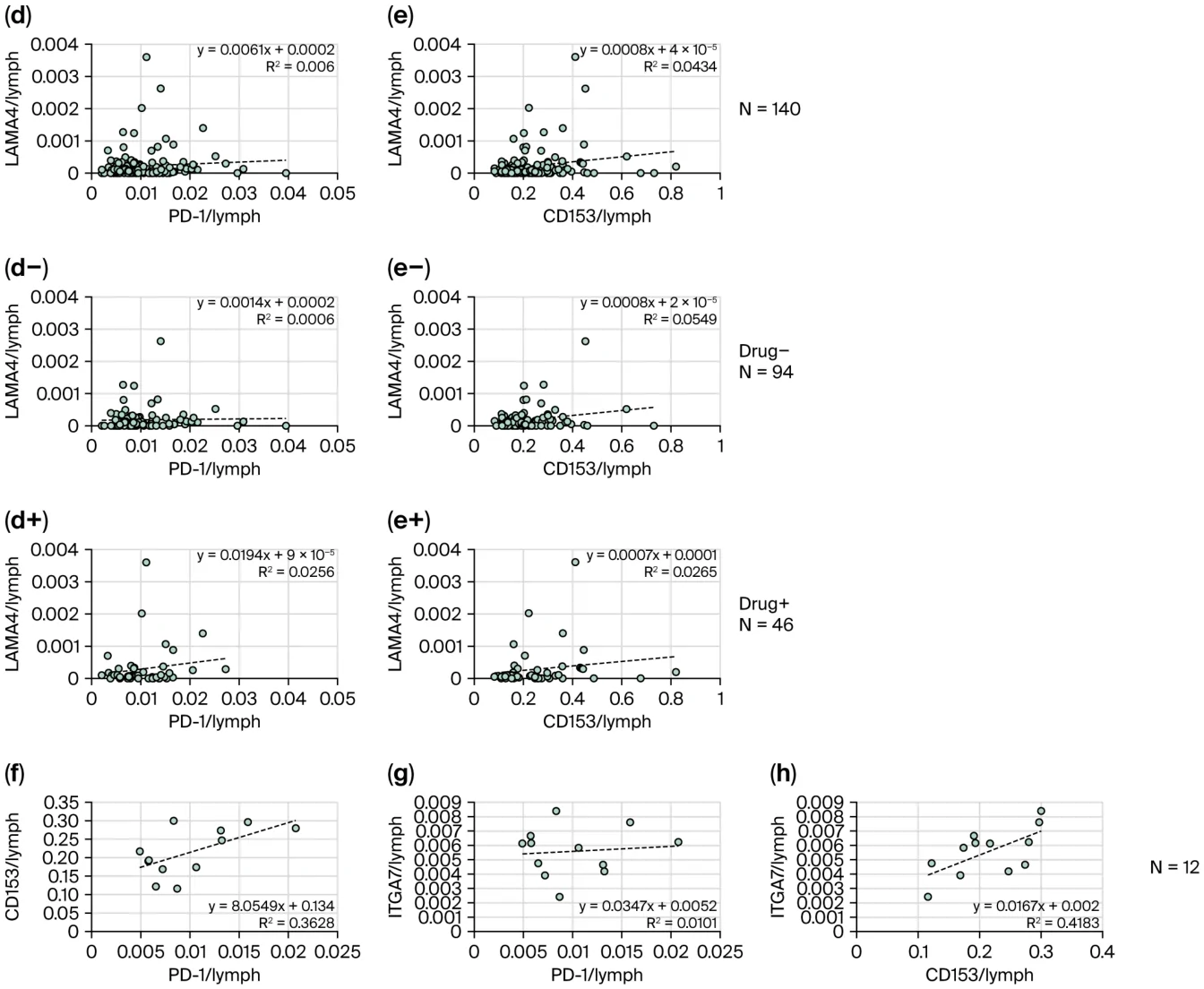
Prospective Cohort Study. A prospective cohort study was conducted in 140 patients (108 females, 32 males) diagnosed with autoimmune diseases at their first outpatient visit to Hyogo Medical University. Of these, 94 patients (71 females, 23 males) were not receiving medication, and 46 patients (37 females, 9 males) were receiving medication. Gene expression levels of senescence- and adhesion-related molecules, *PD-1* (*hPD-1*), *hCD153*, *hITGα7*, and *hLAMα4*, were quantified in leukocyte fractions using TaqMan assays. Expression values were normalized to *GAPDH* and expressed as ratios per lymphocyte (Lymph). Correlation analyses were performed between: (**a**,**a−**,**a+**) *hPD-1*/Lymph and *hCD153*/Lymph, (**b**,**b−**,**b+**) *hPD-1*/Lymph and *hITGα7*/Lymph, (**c**,**c−**,**c+**) *hCD153*/Lymph and *hITGα7*/Lymph, (**d**,**d−**,**d+**) *hPD-1*/Lymph and *hLAMα4*/Lymph, and (**e**,**e−**,**e+**) *hCD153*/Lymph and *hLAMα4*/Lymph. Analyses were performed for the entire cohort (**a**–**e**), untreated patients (**a−**–**e−**), and treated patients (**a+**–**e+**). In addition, correlations between (**f**) *hPD-1*/Lymph and *hCD153*/Lymph, (**g**) *hPD-1*/Lymph and *hITGα7*/Lymph, and (**h**) *hCD153*/Lymph and *hITGα7*/Lymph were evaluated in 12 female patients diagnosed with Sjögren’s disease (SjD), including untreated and treated individuals.

Among 140 outpatients, a positive correlation was observed between *hPD-1*/Lymph and *hCD153*/Lymph (R^2^ = 0.26) (Figure 7a). A stronger correlation was observed in 94 untreated patients (R^2^ = 0.42) (Figure 7(a−)), whereas no correlation was detected in 46 treated patients (R^2^ = 0.12) (Figure 7(a+)). Across all 140 patients, no correlation was found between *hPD-1*/Lymph and *hITGα7*/Lymph (R^2^ = 0.10) (Figure 7b). Similarly, no correlation was observed in the untreated (R^2^ = 0.15) or treated patients (R^2^ = 0.02) (Figure 7(b−,b+)). In contrast, a positive correlation was observed between *hCD153*/Lymph and *hITGα7*/Lymph in the full cohort (R^2^ = 0.43) (Figure 7c). This correlation persisted in untreated patients (R^2^ = 0.31) (Figure 7(c−)) and treated patients (R^2^ = 0.61) (Figure 7(c+)). Conversely, no correlation was observed between *hPD-1*/Lymph or *hCD153*/Lymph and *hLAMα4*/Lymph in the full cohort (R^2^ = 0.00 and R^2^ = 0.04) (Figure 7d,e), or in the untreated or treated subgroups (R^2^ = 0.00 and 0.05; R^2^ = 0.02 and 0.02) (Figure 7(d+,d−,e+,e−)).

In 12 patients with SjD, a positive correlation was observed between *hPD-1*/Lymph and *hCD153*/Lymph (R^2^ = 0.36) (Figure 7f). No correlation was found between *hPD-1*/Lymph and *hITGα7*/Lymph (R^2^ = 0.01) (Figure 7g), whereas a positive correlation was observed between *hCD153*/Lymph and *hITGα7*/Lymph (R^2^ = 0.41) (Figure 7h).

These findings suggest that hITGα7 is expressed on CD153-expressing hSA-Tfh cells in patients with various autoimmune diseases, including SjD, who are undergoing pharmacological therapy.

### 2.9. Retrospective Cohort Study Using Labial Gland Biopsies

**Figure 8:**
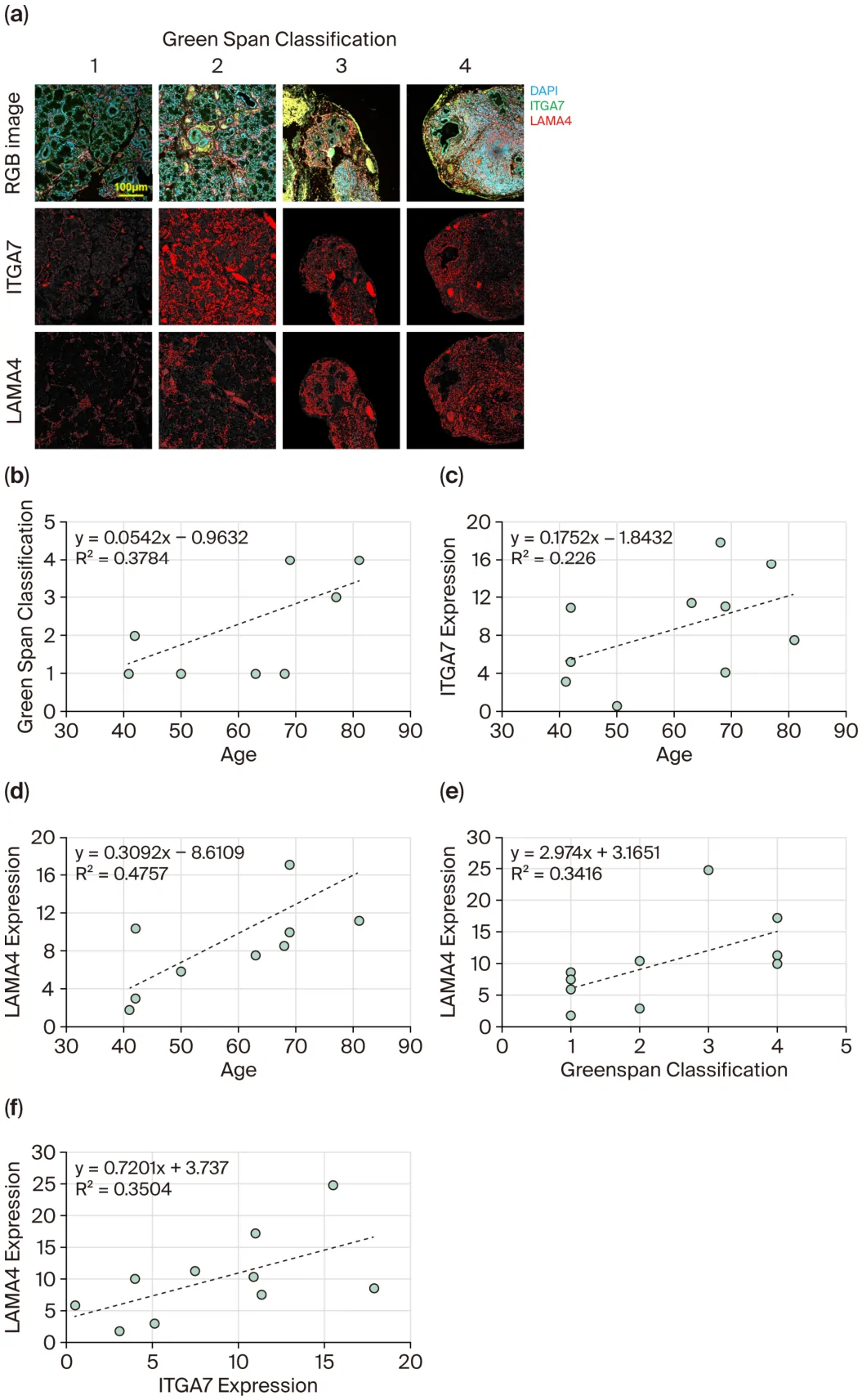
Retrospective Cohort Study in Patients with Sjögren’s Disease. A retrospective cohort study was conducted in 10 female patients diagnosed with Sjögren’s disease (SjD) at their first outpatient visit to Hyogo Medical University. According to the Greenspan (GS) classification, the cohort included four GS1, two GS2, one GS3, and three GS4 cases. (**a**) Paraffin-embedded labial salivary gland sections were incubated with primary antibodies, anti-ITGα7 mIgG (E-2) and anti-LAMα4 rabbit IgG (6C3) at 4 °C for 24 h. Sections were then incubated at 22 °C for 2 h with Alexa Fluor™ 488-conjugated anti-mouse IgG and Alexa Fluor™ 647-conjugated anti-rabbit IgG secondary antibodies, together with DAPI. RGB images were acquired using a Zeiss LSM780 confocal laser scanning microscope. Double-positive cells were quantified using NIH ImageJ (RawIntDen). Platelet aggregates resulting from hemorrhage also stain a pale yellowish-green. Correlation analyses were performed between (**b**) age and GS classification, (**c**) age and hITGα7 expression, (**d**) age and hLAMα4 expression, (**e**) GS classification and hLAMα4 expression, and (**f**) hITGα7 expression and hLAMα4 expression.

A retrospective analysis of paraffin-embedded labial gland biopsies from SjD patients was conducted to examine the roles of hITGα7 and hLAMα4 (Figure 8a). GS1 (mild infiltration): hITGα7 was expressed in cells adhering to the vascular endothelium, and hLAMα4 was expressed in endothelial cells and the basement membrane of periductal edema. GS2 (no follicles containing >50 cells): hITGα7 and/or hLAMα4 were expressed in endothelial-adherent cells, and hLAMα4 was also expressed in endothelial cells and periductal basement membranes. GS3 (one follicle containing >50 cells): hITGα7 and/or hLAMα4 were expressed in endothelial-adherent cells and some periductal follicles. hLAMα4 was also expressed in endothelial cells, basement membranes, and infiltrating cells within the periductal edema. GS4 (more than 1 follicle containing >50 cells): hITGα7 and/or hLAMα4 were expressed in endothelial-adherent cells and in follicular structures within the periductal edema. Notably, numbers of hLAMα4-expressing cells were increased with follicle enlargement.

A positive correlation was observed between GS classification and age (R^2^ = 0.37) (Figure 8b). Age also correlated positively with hITGα7 (R^2^ = 0.22) and hLAMα4 expression (R^2^ = 0.47) (Figure 8c,d). Furthermore, GS classification correlated with hLAMα4 expression per unit area (R^2^ = 0.34) (Figure 8e), and hITGα7 expression per unit area correlated with hLAMα4 expression (R^2^ = 0.35) (Figure 8f).

These findings suggest that in patients with Sjögren’s disease (SjD), hITGα7 on hSA-Tfh cells interacts with hLAMα4 within the salivary gland microenvironment, potentially contributing to the formation of tertiary lymphoid structures (TLS) and disease progression.

## 3. Discussion

ITGα7 contains two extracellular isoforms generated by the alternative selection of exon 5 or exon 6 and has at least three cytoplasmic splice variants [16]. Although isoform-specific interactions between ITGα7 and LAMs have been implicated in the regulation of adhesion, differentiation, and motility, the underlying mechanisms remain incompletely defined. In this study, we identified a previously uncharacterized *mITGα7 isoform 2* that selectively utilizes exon 5 in mSA-Tfh cells from the spleens of 36-week-old female NI mice (Figure 1b and Figure 2d). These findings suggest that hITGα7 isoform 1, which also incorporates exon 5, is expressed in hSA-Tfh cells in the spleens of SjD patients.

Our findings further underscore the complexity of ITGα7-LAM interactions. The comprehensive profiling of ITGα7 isoforms in mSA-Tfh cells within the salivary gland microenvironment of NI and mITGα7-deficient mice, as well as in SjD patients, will be essential to clarify isoform-specific functions (Figure 2b,c). Similar analyses of human chronic myeloid leukemia (hK562) cells (Figure 2e) together with ITGβ subunit profiling will also be required. Nevertheless, our current data indicate that ITGα7β1 selectively binds to LAMα1/α2 in an isoform-independent manner.

Downstream signaling events triggered by ITGα7β1-LAM engagement remain poorly understood. In macrophages derived from primary monocytes or human acute monocytic leukemia (hTHP-1) cells, hITGα7 interacts with LAMα2 to activate phosphoinositide 3-kinase (PI3K)/protein kinase B (AKT) signaling and induce dendritic cell-like differentiation, accompanied by reduced PI3K-p85α expression following granulocyte-macrophage colony-stimulating factor (GM-CSF) stimulation [15]. Conversely, mITGα7-deficient mice exhibited a dystrophic-like phenotype characterized by elevated heat shock protein (Hsp) 72/73 and c-Jun N-terminal kinase (JNK) 2 activity, driven by focal adhesion kinase (FAK) activation and sustained c-Raf-1/mitogen-activated protein kinase (MAP2K) signaling [17]. Consistent with these findings, our experiments demonstrated the selective activation of ITGα7 by LAMα2 in mC2C12 cells (Figure 3b), as well as a complex signaling circuit in which OPN production is regulated through ERK activation and RhoA suppression (Figure 3). Together, these results suggest that ITGα7-LAMα2 interactions constitute a critical regulatory axis within the salivary gland vascular niche that shapes mSA-Tfh function and TLS formation in female NI mice.

CD153 has recently been identified as a marker of SASP-producing immune cells, including OPN-producing senescent cells associated with chronic inflammation [18]. A B-cell subset capable of producing autoantibodies upon interaction with SA-T cells was shown to express ITGαMβ2 (CD11b) and ITGαXβ2 (CD11c) [19]. In our study, we newly identified ITGαMβ2 expression in CD8^+^/PD-1^+^ cells (Table 1). These findings suggest that, in addition to anti-apoptotic and proliferative signaling mediated by CD30 in age-associated B cells (ABCs) interacting with CD153 on SA-T cells, ITGαMβ2 on SA-T cells may facilitate adhesion to the Leu-Asp-Val (LDV) motif on ABCs.

To further characterize integrin usage, we estimated integrin expression in mSA-Tfh cells using the formula αXβY = αX × βY (relative to CD4^+^/PD-1^+^ cells). mSA-Tfh cells exhibited a relatively high expression of ITGαXβ2 (×9.49) and ITGαLβ2 (×4.68), whereas ITGα6 and ITGα8 were expressed at relatively low levels (Table 1). mSA-B cells had high expressions of ITGαEβ7 (×20.85) and ITGαDβ2 (×5.34) and a low expression of ITGαM. A recent study classified dendritic cell subsets based on ITGαMβ2, ITGαEβ7, and PD-L1 expression [20]. The ITGαMβ2^−^/ITGαEβ7^+^/PD-L1^high^ subset expresses retinaldehyde dehydrogenase 2 in the intestine and induces regulatory T cells to maintain immune homeostasis. This subset declines with age, although the mechanisms involved in immune recovery remain unclear.

We propose that the age-dependent expansion of ITGαEβ7^+^ subsets within CD4^−^ cells (likely mSA-B cells), which localize exclusively to B-cell follicles, and within CD4^+^ cells (likely mSA-Tfh cells), which are distributed across T-cell zones and B-cell follicles, collectively contributes to SA-dependent TLS formation in aged female NI mice (Table 1 and Supplementary Figure S3a,b). This remodeling of ITG-defined immune niches may ultimately drive the development of SjD.

The conditional deletion of LAMα4 in fibroblastic reticular cells (Pdgfrb-Cre × LAMα4^fl/fl^) further revealed that LAMα4 preserved the high endothelial venule and ductal architecture, supported T-cell homing, suppressed alloantigen-specific T-cell activation, promoted regulatory T-cell and dendritic cell differentiation, and contributed to a tolerogenic lymph node niche [21]. Our findings are consistent with these observations. First, the adhesion of mSA-Tfh cells to LAMα2/α4 was inhibited by anti-ITGα7 mIgG (E-2) (Figure 2a,b). Second, mSA-Tfh cells from mITGα7-deficient mice exhibited reduced adhesion to LAMα1/α2 (Figure 2c). Third, hK562 cells overexpressing hITGα7 isoform 2 selectively adhered to LAMα1/α2 (Figure 2e). Together, these results indicate that hSA-Tfh cells expressing hITGα7 isoform 1 and mSA-Tfh cells expressing mITGα7 isoform 2 interact with LAMα2/α4 within organ-specific vascular niches [22,23].

The administration of anti-ITGα7 mIgG (E-2), which lacks ADCC activity (Supplementary Figure S1), to 24-week-old female NI mice resulted in the disappearance of mSA-T cells from the thymus, spleen, and submandibular glands within 4 weeks, improving GS4 pathology by approximately 60% (Figure 6). In a prospective cohort of 140 outpatients, *hITGα7*/Lymph expression positively correlated with *hCD153*/Lymph expression (R^2^ = 0.43) (Figure 7a–c) and a similar correlation (R^2^ = 0.41) was observed in 12 SjD patients (Figure 7f–h). In a retrospective cohort of labial gland biopsies from SjD patients, hITGα7 and hLAMα4 were expressed in the vascular endothelium and ductal structures of GS2 patients, and in the follicles and ductal structures of GS3 and GS4 patients (Figure 8a). hITGα7 expression correlated positively with hLAMα4 expression (R^2^ = 0.35). We need further experiments to confirm these speculations.

Collectively, these results support a model in which circulating hITGα7^+^/CD153^+^ SA-immune cells adhere to LAMα4^+^ vascular endothelium within salivary gland niches, promoting TLS formation through interactions with LAMα4^+^ ductal cells and/or LAMα4^+^ SA-B cells. This ITGα7-LAMα4 axis may drive SASP-mediated chronic tissue remodeling and contribute to the pathogenesis of SjD.

## 4. Materials and Methods

### 4.1. Ethical Approval and Consent to Participate

#### 4.1.1. Prospective Cohort Study

The prospective cohort study (n = 140) was approved by the Ethics Committees of Hyogo Medical University, and written informed consent was obtained from all participants prior to enrollment (Table 2). Overall, 108 female patients (mean age 56.85 years) and 32 male patients (mean age 59.12 years) diagnosed with autoimmune diseases at their first outpatient visit to the Department of Diabetes, Endocrinology, and Clinical Immunology at Hyogo Medical University were included in this study. Their ages ranged from 16 to 90 years (overall mean 57.37 years). Among the patients receiving medication, 37 females (16–89 years; mean 57.51 years) and 9 males (20–84 years; mean 63.44 years) were included (overall mean 58.67 years). Among untreated patients, 71 females (16–87 years; mean 56.50 years) and 23 males (17–90 years; mean 57.43 years) were included (overall mean 56.73 years). Two female patients with SjD receiving medication (57–76 years; mean 66.5 years) and ten untreated female SjD patients (19–84 years; mean 66 years) were also enrolled in the study.

The gene expression of senescence- and adhesion-related molecules in leukocyte fractions was analyzed using TaqMan assays. For each outpatient, the expression levels of *human PD-1* (*hPD-1*), *hCD153*, *hITGα7*, and *hLAMα4* were normalized to *GAPDH* and expressed per lymphocyte (Lymph). Correlations were assessed between *hCD153*/Lymph and *hITGα7*/Lymph or *hLAMα4*/Lymph, and between *hPD-1*/Lymph and *hCD153*/Lymph, *hITGα7*/Lymph, or *hLAMα4*/Lymph.

A one-dimensional normality test (Shapiro–Wilk test) was performed on the ITGa7/Lymph data from peripheral blood leukocytes of 12 SjD patients; based on a mean (x) of 0.00533 and a standard deviation (s) of 0.00163, the results were W = 0.94 and *p* = 0.38. Since *p* = 0.38 > 0.05, the data are not considered to differ significantly from a normal distribution.

#### 4.1.2. Retrospective Cohort Study

Two retrospective cohort studies (n = 10) were also approved by the Ethics Committees of Hyogo Medical University, and written informed consent was obtained from all participants (Table 3). Ten female patients diagnosed with SjD at their first outpatient visit (41–81 years; mean 60.2 years) were included. Based on GS grading, four patients were GS1, two were GS2, one was GS3, and three were GS4. Paraffin-embedded labial gland sections were stained with H&E. Red/green/blue (RGB) images were acquired using a BX53 microscope (Olympus, Tokyo, Japan) and Olympus cellSens software version 4.5, and pathological changes were evaluated according to GS classification.

A one-dimensional normality test (Shapiro–Wilk test) was performed on LAMα4 data obtained from paraffin-embedded specimens of 10 SjD patients, using a mean (x) of 10.205 and a standard deviation (s) of 6.41; the results yielded W = 0.82 and *p* = 0.03. The result of *p* = 0.03 (<0.05) indicates that the distribution differs significantly from a normal distribution and exhibits skewness.

For immunofluorescence, paraffin sections were incubated with primary antibodies, including anti-ITGα7 mouse IgG (E-2; Santa Cruz Biotechnology Inc., Paso Robles, CA, USA) and anti-LAMα4 rabbit IgG (6C3; Santa Cruz Biotechnology Inc.) at 4 °C for 24 h. Sections were then incubated at 22 °C for 2 h with Alexa Fluor™ 488-conjugated sheep anti-mouse IgG (H+L) and Alexa Fluor™ 647-conjugated goat anti-rabbit IgG (H+L) (Jackson ImmunoResearch Lab. Inc., West Grove, PA, USA). Confocal images were acquired using an LSM780 microscope (Zeiss, Tokyo, Japan). Double-positive cells were quantified using NIH ImageJ software (RawIntDen).

#### 4.1.3. Animal Study

NI mice and mITGα7 KO mice were maintained under specific pathogen-free conditions at the Pathogenesis Model Research Center, Hyogo Medical University. All animal procedures (approval no. 25-074AG) and genetic experiments (approval no. 24-049G) were conducted in accordance with institutional guidelines and approved by the Animal Experimentation Committee of Hyogo Medical University. Experiments using B6 mice are typically conducted between 8 and 12 weeks of age. It was reported that in the spleens of B6 male mice, the proportion of PD-1-positive SA-immune cells while showing no significant difference at 24 weeks of age increases significantly to approximately 10% by 48 weeks of age [4]. Consequently, the analysis of SA-immune cells in genetically modified mice on a B6 background was performed at 15 weeks of age, a time point between 12 and 24 weeks. Meanwhile, it was reported that NI female mice exhibit a significant increase in splenic PD-1-positive SA-immune cells at 24 weeks of age compared to B6 female mice, as well as a decrease in salivary secretion by 36 weeks of age [5]. Therefore, antibody administration experiments involving NI female mice were conducted at 24 weeks of age, the stage at which cellular infiltration into the submandibular glands is observed.

### 4.2. ITGα7 and LAMα4 KO Mice

Guide RNAs (gRNAs) targeting *intron 1* and *intron 3* of the *ITGα7* gene, and *intron 2* and *intron 3* of the *LAMα4* gene, were designed using CRISPRdirect [24]. The gRNA sequences were as follows: *ITGα7-1*, *5*′*-gccctgaggagttgaggcca-3*′; *ITGα7-2*, *5*′*-agcaccttcagaatactaac-3*′; *LAMα4-1*, *5*′*-tgtttgtttgaagtaagcta-3*′; *and LAMα4-2*, *5*′*-tttagctctatgcatagttc-3*′. The corresponding crRNA, tracrRNA, and Cas9 nuclease were purchased from Integrated DNA Technologies (Coralville, IA, USA). Male and female C57BL/6 mice (CLEA Japan, Tokyo, Japan) were used as sperm and oocyte donors. A mixture containing 200 ng/µL Cas9 protein and 6 pmol/µL gRNA was introduced into in vitro-fertilized zygotes by electroporation using an NEPA21 super electroporator (Nepa Gene, Chiba, Japan) with a 1 mm gap electrode (CUY501P1-1.5).

### 4.3. Antibodies

E-2 is a mouse monoclonal antibody raised against amino acids 878–917 within the extracellular domain of hITGα7. This region shares 90% homology with amino acids 1018–1057 in mice. 012-Z is a mouse monoclonal antibody generated against recombinant hITGα7 (Santa Cruz Biotechnology Inc.). G-12 is a mouse monoclonal antibody raised against amino acids 1856–2099 within an internal region of hLAMα1 (Santa Cruz Biotechnology Inc.), which exhibits 64% homology with the corresponding region in mice. 4H8-2 is a rat monoclonal antibody generated against native mouse LAMα2, with its epitope mapped to the N-terminal domain (Santa Cruz Biotechnology Inc.). B-4 is a mouse monoclonal antibody raised against amino acids 2811–2940 near the C-terminus of hLAMα2 (Santa Cruz Biotechnology Inc.). This region has 89% homology with amino acids 2807–2935 in mice. 6C3 is a rabbit monoclonal antibody raised against purified hLAMα4β1γ1 isolated from platelets.

### 4.4. FACS Analysis

Bone marrow cells were stained with Brilliant Violet 421™ anti-mouse Lineage Cocktail, APC anti-mouse CD34, FITC anti-mouse Ly-6A/E (Sca-1), and PE anti-mouse CD117 (c-Kit) antibodies (all from BioLegend, San Diego, CA, USA) in 500 µL PBS containing 2% FBS (Thermo Fisher Scientific, Waltham, MA, USA) and 0.5 µM EDTA (Nacalai Tesque Inc., Kyoto, Japan). Lineage^−^/CD34^−^/c-Kit^+^/Sca-1^+^ stem cells were analyzed on a FACSAria III (BD Biosciences, East Rutherford, NJ, USA).

Peripheral blood erythrocytes collected in heparin sodium (1000 U/mL; Motida Inc., Tokyo, Japan) were lysed using BD Pharm Lyse™ buffer (BD Biosciences). Homogenized thymic cells and leukocytes were stained with FITC anti-mouse CD4, Brilliant Violet 421™ anti-mouse PD-1, and APC anti-mouse CD153 antibodies (BioLegend). CD4^+^/PD-1^+^ and CD4^+^/PD-1^+^/CD153^+^ cells were sorted using a FACSAria III machine.

Similarly, splenic cells prepared in the same buffer were stained with FITC anti-mouse CD4, PE anti-mouse/human CD45R/B220, Brilliant Violet 421™ anti-mouse PD-1, and APC anti-mouse CD153 antibodies. CD4^+^/PD-1^+^/CD153^−^ cells, CD4^+^/PD-1^+^/CD153^+^ (mSA-Tfh) cells, B220^+^/PD-1^−^ cells, and B220^+^/PD-1^+^ (mSA-B) cells were collected by a FACSAria III machine.

For T-cell subset analysis, cells were stained with APC anti-mouse CCR7 (BioLegend), PE anti-rat CD45RA (BioLegend), and FITC anti-mouse CD4 antibodies. CCR7^−^/CD45RA^−^ effector memory T cells (Tem), CD4^+^ Tem cells, CCR7^−^/CD45RA^+^ T effector memory RA^+^ cells (Temra) cells, and CD4^+^ Temra cells were sorted using a FACSAria III machine.

### 4.5. Sandwich ELISA

Spleen cells from 36-week-old female NI mice were stained with FITC anti-mouse CD4, PE anti-human/mouse CD45R/B220, APC anti-mouse CD153, and Brilliant Violet 421™ anti-mouse PD-1 antibodies in 500 µL PBS containing 2% FBS and 0.5 µM EDTA. mSA-Tfh cells and mSA-B cells were sorted using a FACSAria III machine.

A total of 2 × 10^4^ mSA-Tfh cells and/or mSA-B cells were cultured in 96-well U-bottom plates (Corning Inc., Steuben County, NY, USA) in 200 µL DMEM supplemented with 10% FBS and penicillin/streptomycin (Thermo Fisher Scientific). After 48 h, concentrations of OPN and IFN-γ in culture supernatants were quantified using the Mouse Osteopontin ELISA Kit (Sigma-Aldrich, Darmstadt, Germany) and Mouse IFN-γ ELISA Kit (Proteintech Group, Tokyo, Japan), according to the manufacturers’ instructions.

### 4.6. PCR Analysis

DNA from mouse and human cells was extracted using the SimplePrep™ reagent for DNA (Takara Bio Inc., Kyoto, Japan) and used as a template for the amplification of *ITGα7 isoform 1* and *isoform 2* with the TaKaRa PCR Amplification Kit (Takara Bio Inc.). For *mITGα7*, the S13-F primer (GGTTCTGTCAGCAGGGCACA) and S13-R primer (CAAAGGGGCTGTGGTCATT) amplified a 306 bp product corresponding to *isoform 1* and a 318 bp product corresponding to *isoform 2*. For *hITGα7*, the S12-F primer (CACTACCTCCTCTTTGGGGC) and S12-R primer (AGCCACAAAGCTCAGCTCTT) amplified a 234 bp *isoform 1* product and a 222 bp *isoform 2* product. PCR products were resolved on 2.5% PrimeGel™ Agarose PCR-Sieve gels (Takara Bio Inc.) to visualize isoform-specific band sizes.

### 4.7. Cell Binding Assay

A 96-well flat-bottom culture plate (Corning Inc.) was pre-coated with 200 μL of PBS or one of the following laminin E8 fragments (final concentration 0.155 μg/mL): iMatrix-111 (LAMα1/β1/γ1; predicted to bind to ITGα7β1 or ITGα6β1), iMatrix-221 (LAMα2/β2/γ1; predicted to bind to ITGα7β1), iMatrix-332 (LAMα3/β3/γ2; predicted to bind to ITGα3β1 or ITGα6β4), iMatrix-411 (LAMα4/β1/γ1; predicted to bind to ITGα6β1), or iMatrix-511 (LAMα5/β1/γ1; predicted to bind to ITGα6β1) (Nippi Inc., Tokyo, Japan). Plates were incubated for 1 h at 37 °C in a 5% CO_2_ incubator (MCO-50AIC, PHC, Osaka, Japan) and then washed twice with PBS.

Cells (1 × 10^3^ per well) were seeded in 100 μL of culture medium and incubated for 10 or 30 min. After incubation, wells were washed once with PBS, followed by the addition of 100 μL of culture medium and 10 μL of WST-8 reagent (Cell Counting Kit-8, Dojindo, Kumamoto, Japan). After 2 h, the number of adherent cells was quantified by measuring absorbance at 450 nm using a SPECTRAmax PLUS384 microplate reader (Molecular Devices Japan, Tokyo, Japan).

### 4.8. Echocardiographic Measurements

For echocardiographic assessment, mice were anesthetized with 2% isoflurane (Fujifilm Wako Chemicals, Osaka, Japan) and placed on a heated platform. Cardiac function including left ventricular end-diastolic diameter, end-systolic diameter, and fractional shortening was evaluated using a high-frequency ultrasound system (Vevo 2100 with MS400 transducer; FUJIFILM VisualSonics, Toronto, ON, Canada). Two-dimensional parasternal short-axis images were obtained at the level of the papillary muscles, and M-mode tracings were recorded for quantitative analysis.

### 4.9. Transfection

A total of 1 × 10^5^ chronic myeloid leukemia hK562 cells (RCB1635, Riken Bioresource Center, Ibaraki, Japan) were seeded in 100 μL of culture medium in a 96-well U-bottom plate and incubated with lentivirus (MOI 10; pLV[Exp]-Puro-EF1A > hITGα7 isoform 2, VectorBuilder, Chicago, IL, USA) for 6 h. The cells were then transferred to a 6-well plate (Corning Inc.) containing 3 mL of culture medium. After 2 days, the cells were washed twice with 10 mL of PBS and resuspended in 100 mL of culture medium supplemented with puromycin (Thermo Fisher Scientific). Subsequently, 100 cells per 100 μL were seeded into 96-well flat-bottom plates. After 2 weeks, wells containing single-cell-derived colonies were expanded from 24-well to 6-well plates. Clones exhibiting an increased expression of *hITGα7 isoform 2* relative to *GAPDH* were selected by qPCR as described previously.

For mC2C12 myoblasts, 1 × 10^5^ cells (RCB0987, Riken Bioresource Center, Tsukuba, Ibaraki, Japan) were seeded in 2 mL of culture medium in 6-well plates and transfected with Lipofectamine (Thermo Fisher Scientific) together with a pGL4.33 [luc2P/SRE/Hygro] vector or a pGL4.34 [luc2P/SRF-RE/Hygro] vector. These vectors contain serum response elements (SRE or SRF-RE) that drive luc2P luciferase expression in response to mitogen-activated protein kinase (MAPK)/extracellular signal-regulated kinase (ERK) or RhoA pathway activation, respectively. After 6 h of incubation, the transfection mixture was replaced with 3 mL of fresh culture medium. Two days later, the cells were washed twice with 10 mL of PBS and resuspended in 100 mL of culture medium containing Geneticin™ (Thermo Fisher Scientific). The cells were then seeded at 100 cells per 100 μL into 96-well flat-bottom plates. After 2 weeks, wells containing single-cell-derived colonies were expanded from 24-well to 6-well plates. Stable clones, designated 1A-9 and 5F-4, were selected based on luciferase activity in the presence of 20% FBS.

### 4.10. Cell Signaling Assay

A total of 2 × 10^4^ 1A-9 or 5F-4 cells were seeded in 40 μL of culture medium into a 96-well flat-bottom white plate and incubated at 37 °C in a 5% CO_2_ atmosphere. After 24 h, 10 μL of anti-ITGα7 mIgG (E-2) was added at final concentrations ranging from 0 to 1 μg/mL. Following a 30 min incubation, 10 μL of iMatrix-221, iMatrix-411, or iMatrix-511 was added to achieve a final concentration of 10 μg/mL. After an additional 2 h incubation, 40 μL of OneGlo medium (Promega, Madison, WI, USA) was added. Following a further 2 h incubation, MAPK/ERK or RhoA pathway activation was quantified as luciferase activity using a GloMax 96 luminometer (Promega).

### 4.11. ADCC Assay

ADCC assays were performed using the ADCC Reporter Bioassay Complete Kit (Raji) (Promega). A total of 1 × 10^4^ ADCC bioassay target cells (Raji) or mSA-Tfh cells were seeded in 40 μL of Opti-MEM medium (Thermo Fisher Scientific) containing 1% FBS in a 96-well flat-bottom white plate. Subsequently, 10 μL of mIgG, anti-CD20 antibody, or anti-ITGα7 mIgG (E-2) was added to achieve final concentrations of 0–10 μg/mL, and the cells were incubated at 37 °C in a 5% CO_2_ incubator. After 30 min, effector cells (six-fold excess relative to target cells) expressing CD20 were added in 40 μL of Opti-MEM containing 1% FBS. Following a 6 h incubation, 60 μL of Bio-Glo™ Luciferase Assay Reagent was added and incubated for 30 min. ADCC activity was quantified as luciferase activity using a GloMax 96 luminometer.

### 4.12. In Vivo Assay

NI mice were maintained on a standard diet until 24 weeks of age. Mice received intraperitoneal injections of mIgG (n = 5) or anti-ITGα7 mIgG (E-2; 10 μg/200 μL, n = 5) once daily for 3 consecutive days at 24 and 25 weeks of age. The bone marrow, thymus, spleen, and submandibular glands were collected at 28 weeks of age.

The bone marrow, thymus, and spleen cells were processed and stained using identical procedures. In the bone marrow, the proportions of CD34^+^/lineage^−^ cells and CD34^+^/lineage^−^/c-Kit^+^/Sca-1^+^ stem cells were analyzed. In the thymus, CD4^+^/PD-1^−^ cells, CD4^+^/PD-1^+^ cells, and mSA-Tfh cells were quantified. In the spleen, CD4^+^/PD-1^+^ cells, mSA-Tfh cells, B220^+^/PD-1^−^ cells, mSA-B cells, Tem cells, CD4^+^ Tem cells, Temra cells, and CD4^+^ Temra cells were analyzed. All flow cytometric analyses were performed using a FACSAria III machine.

Submandibular glands and spleens were fixed in formalin for 24 h, embedded in paraffin, and sectioned at 2 μm thickness. Sections were stored at 4 °C until staining was performed. To evaluate cellular infiltration into the submandibular glands and the cell density in the spleen, sections were stained with H&E. RGB images were acquired using BX53 microscope. Pathological changes in cellular infiltration were assessed using the previously described GS classification [5]. Spleen cell density (RawIntDen) was quantified using NIH ImageJ software.

For immunofluorescence staining, paraffin sections were incubated at 4 °C for 24 h with primary antibodies, including anti-ITGα7 mIgG (E-2) and anti-CD153 rabbit IgG (Santa Cruz Biotechnology Inc.). Sections were then incubated at 22 °C for 2 h with Alexa Fluor™ 488-conjugated sheep anti-mouse IgG (H+L) and Alexa Fluor™ 647-conjugated goat anti-rabbit IgG (H+L) secondary antibodies, together with DAPI (Merck, Land Hessen, Germany). RGB images were captured using an LSM780 confocal laser scanning microscope. The number of double-positive cells (RawIntDen) was quantified using NIH ImageJ software.

### 4.13. Statistical Analysis

For the assessment of statistical significance in in vitro experiments, results from three independent experiments each comprising at least three samples prepared from the same starting material were analyzed using KyPlot 6.0.2; a parametric Tukey test followed by multiple comparison analysis was performed. For the assessment of statistical significance in histopathological evaluations, RGB images acquired via microscopy were analyzed using Fiji ImageJ and KyPlot 6.0.2; a non-parametric Wilcoxon test followed by multiple comparison analysis was conducted in accordance with previously reported methods [5]. Statistical significance was defined as * *p* < 0.05, ** *p* < 0.01, and *** *p* < 0.001.

In our laboratory’s FACS analyses, statistical analysis for conditions with a sample size of 10 or fewer is typically performed after excluding the maximum and minimum values. This pre-established protocol is applied regardless of the results. In the antibody administration study, blinding is not implemented during the antibody administration and necropsy phases. However, as collected specimens are assigned identification numbers and paraffin-embedded organ sections are prepared by specialized technicians, the immunohistochemical samples are effectively blinded. Nevertheless, an inherent limitation in the blinding process exists in that the person performing the necropsy and the person conducting the microscopic analysis are the same individual.

## 5. Conclusions

Circulating mITGα7^+^ SA-Tfh cells adhere to the mLAMα4^+^ microenvironment of the submandibular gland, thereby promoting TLS formation in female NI mice.

Sex as a biological variable. SjD predominantly affects females. Furthermore, compared with male NI mice, SA-T cells infiltrate the submandibular gland tissue at an earlier stage in female NI mice. This study examined male and female patients as well as male and female mice, revealing findings that differed between the sexes.

## Figures and Tables

**Table 1:** PD-1-induced fold expression of ITGα and/or β in senescence-associated immune cells.

**Up**	**Fold Expression of ITGα or β Induced by PD-1**																	
	**α3**	**α5**	**α7**	**α9**	**α11**	**αD**	**αE**	**αL**	**αM**	**αV**	**αX**	**β1**	**β2**	**β3**	**β4**	**β5**	**β7**	**β8**
CD4	2.62			3.71	4.04	6.30	4.83			2.41		3.71				4.00		11.43
CD153								2.07			4.20		2.26				2.61	
CD8		2.78	16.22	3.70		3.65			14.30					2.42	3.42	36.72		3.70
F4/80	2.75		2.17	10.40	2.17	4.69									2.36			
B220						2.52	5.65						2.12				3.69	2.53
**Down**	**Fold Expression of ITGα or β Induced by PD-1**																	
	**α1**		**α2**	**α4**	**α6**		**α8**		**αE**	**αM**		**αX**	**β2**	**β3**		**β4**		**β6**
CD4	10.19				2.84					2.99		5.50		4.47		2.97		
CD153					2.77		2.99											
CD8	2.99				3.30													
F4/80	5.79		24.16	2.24			2.81		3.14	4.03		7.16	2.36					2.05
B220										2.88								
**Calc**	Fold Expression of ITGα*β* Induced by PD-1																	
	**α3β1**		**α9β1**		**α11β1**		**αDβ2**		**αEβ7**	**αLβ2**		**αVβ1**	**αVβ5**	**αVβ8**		**αXβ2**		
CD4	2.62 × 3.71		3.71 × 3.71		4.04 × 3.71		6.30		4.83			2.41 × 3.71	2.41 × 4.00	2.41 × 11.43				
CD153										2.07 × 2.26						4.20 × 2.26		
B220							2.52 × 2.12		5.65 × 3.69									
**Total**	**Fold Expression of ITGαβ Induced by PD-1**																	
	**α3β1**		**α9β1**		**α11β1**		**αDβ2**		**αEβ7**	**αLβ2**		**αVβ1**	**αVβ5**	**αVβ8**		**αXβ2**		
CD4	9.72		13.76		14.99		6.30		4.83			8.94	9.64	27.55				
CD153										4.68						9.49		
B220							5.34		20.85									

* represents the gene expression rates of α and β, respectively.

**Table 2:** ITGα7 expression per lymphocyte percentage of outpatients at the Division of Diabetes, Endocrinology and Clinical Immunology, Department of Internal Medicine, Hyogo Medical University.

Pt. Number	Sex	Age	Disease	Treatment	Lymphocytes (%)	PD1/ Lymph	CD153/ Lymph	LAMA4/ Lymph	ITGA7/ Lymph
2	M	84	Late-onset rheumatoid arthritis	Drug (+)	18.1	0.022654452	0.359967459	0.001396410	0.009657988
3	F	69	Osteoarthritis	Drug (−)	16.9	0.014032393	0.452159866	0.002622039	0.012823242
4	M	51	Autoinflammatory syndrome	Drug (+)	35.3	0.015116416	0.159569841	0.001062928	0.001812579
5	F	74	Systemic Sclerosis	Drug (+)	34.2	0.010222783	0.221892340	0.002019105	0.003515466
6	F	32	Rheumatoid arthritis	Drug (−)	9.1	0.039499813	0.731023477	0.000000000	0.016378984
7	F	57	Susp	Drug (−)	22.6	0.012221840	0.272741345	0.000000000	0.005394127
11	M	53	Fibromyalgia	Drug (−)	22.5	0.008862952	0.252088654	0.000000000	0.008099245
12	F	20	Susp	Drug (+)	21.7	0.009513771	0.152220340	0.000000000	0.003837103
13	M	66	Susp	Drug (+)	19.2	0.007344204	0.246698660	0.000000000	0.010386272
14	M	70	Susp	Drug (−)	21.6	0.003935866	0.201785831	0.000394113	0.005376551
15	F	64	Antiphospholipid syndrome	Drug (−)	38.8	0.008465855	0.311190828	0.000000000	0.010865297
16	F	47	IgG-related diseases	Drug (−)	25.5	0.008617176	0.201860543	0.001237317	0.005967866
17	F	51	Susp	Drug (−)	25.2	0.011747555	0.163629453	0.000000000	0.002241227
18	F	75	Rheumatoid arthritis	Drug (−)	10.5	0.004984066	0.297619048	0.000000000	0.016193277
20	F	68	Osteoarthritis	Drug (−)	38.8	0.01230912	0.167922964	0.000321225	0.003098684
21	F	48	Psoriatic arthritis	Drug (−)	40.5	0.012040225	0.140049252	0.000000000	0.003052075
22	F	84	Osteoarthritis	Drug (−)	34.0	0.008067813	0.123826616	0.000000000	0.003015041
24	M	70	Rheumatoid arthritis	Drug (−)	16.3	0.006421189	0.282643638	0.001268251	0.007796575
32	F	16	Chronic relapsing multiple osteomyelitis	Drug (−)	26.0	0.012076923	0.114884615	0.000055731	0.001635000
33	F	49	Takayasu arteritis	Drug (−)	27.8	0.006953237	0.137913669	0.000130108	0.006136691
34	F	48	Systemic lupus erythematosus	Drug (+)	20.1	0.014129353	0.342636816	0.000110398	0.003053731
35	M	55	Axial spondyloarthritis	Drug (−)	35.4	0.007776836	0.123135593	0.000000000	0.000948588
36	F	51	Rheumatoid arthritis	Drug (−)	29.4	0.007955782	0.122108844	0.000301837	0.001276190
37	F	51	Rheumatoid arthritis	Drug (−)	42.0	0.002102857	0.121285714	0.000000000	0.000000000
38	F	33	Eosinophilic granulomatosis with polyangiitis	Drug (+)	11.9	0.020630252	0.256302521	0.000259160	0.005157983
39	F	30	Psoriatic arthritis	Drug (−)	29.2	0.016815068	0.095445205	0.000042295	0.022732877
40	M	49	Susp	Drug (−)	15.5	0.021561290	0.371032258	0.000105871	0.002621290
42	F	61	Polyarteritis nodosa	Drug (−)	22.5	0.009800000	0.360222222	0.000267511	0.021897778
44	F	68	Rheumatoid arthritis	Drug (+)	14.4	0.008736111	0.431041667	0.000334861	0.021881944
45	F	83	Sjögren disease	Drug (−)	21.8	0.006513761	0.122201835	0.000114495	0.004752294
46	M	64	Rheumatoid arthritis	Drug (+)	46.8	0.005760684	0.109615385	0.000000000	0.002542735
47	F	71	Sjögren disease	Drug (−)	27.9	0.005745520	0.191003584	0.000000000	0.006670251
48	F	68	Polymyalgia rheumatica	Drug (+)	7.0	0.015314286	0.676714286	0.000000000	0.021514286
49	M	30	Susp	Drug (−)	22.5	0.009466667	0.112800000	0.000244489	0.007275556
50	M	82	Susp	Drug (−)	23.0	0.006778261	0.190826087	0.000102652	0.006130435
51	F	81	Rheumatoid arthritis	Drug (+)	12.6	0.016611111	0.445476190	0.000882540	0.020380952
52	M	76	Polymyalgia rheumatica	Drug (+)	10.5	0.004147619	0.232666667	0.000098191	0.009046667
53	F	65	Rheumatoid arthritis	Drug (+)	15.5	0.003878065	0.305612903	0.000000000	0.007212903
54	F	28	Susp	Drug (+)	44.2	0.002075792	0.132850679	0.000100747	0.003049774
55	F	78	Rheumatoid arthritis	Drug (−)	27.4	0.005437956	0.139927007	0.000000000	0.003454745
56	F	58	Susp	Drug (−)	29.4	0.007632653	0.173265306	0.000262789	0.004489796
57	F	46	Rheumatoid arthritis	Drug (+)	27.9	0.008125448	0.330250896	0.000084337	0.006928315
58	M	48	Behçet’s disease	Drug (−)	31.4	0.005378981	0.191592357	0.000133217	0.004697452
59	F	46	Seronegative RAr/o	Drug (−)	24.7	0.004433198	0.204817814	0.000000000	0.004093117
60	M	73	Dermatomyositis	Drug (+)	13.7	0.008598540	0.436131387	0.000309635	0.007357664
61	M	48	Axial spondyloarthritis	Drug (−)	32.2	0.008972050	0.154409938	0.000256242	0.003400621
62	F	53	Behçet’s disease	Drug (+)	22.8	0.013820175	0.274122807	0.000000000	0.003741667
63	F	78	Susp	Drug (−)	10.7	0.025196262	0.619532710	0.000515888	0.015037383
64	F	55	Relapsing polychondritis	Drug (−)	22.6	0.010137168	0.203141593	0.000129071	0.005902655
65	F	44	Susp	Drug (+)	19.1	0.013774869	0.256753927	0.000000000	0.006701571
66	M	45	Rheumatoid arthritis	Drug (−)	25.6	0.006089844	0.247539063	0.000148281	0.004382813
67	F	18	Susp	Drug (−)	35.0	0.007622857	0.082457143	0.000142629	0.001939143
68	F	75	Susp	Drug (−)	37.1	0.002605121	0.089353100	0.000000000	0.003894879
70	F	78	Polymyalgia rheumatica	Drug (+)	15.9	0.011144654	0.411194969	0.003601887	0.018685535
71	F	72	Rheumatoid arthritis	Drug (+)	16.6	0.005507831	0.176265060	0.00030494	0.010313253
72	M	35	Systemic lupus erythematosus	Drug (−)	22.6	0.006132743	0.193539823	0.000337124	0.003497788
73	F	72	Rheumatoid arthritis	Drug (+)	31.6	0.003313291	0.206170886	0.000701266	0.004401899
74	F	57	Susp	Drug (−)	34.1	0.007557185	0.199882698	0.000000000	0.003466276
79	F	30	Autoinflammatory syndrome	Drug (+)	39.9	0.007576441	0.175012531	0.000058772	0.003448622
80	F	79	Rheumatoid arthritis	Drug (−)	19.8	0.010500000	0.204696970	0.000077071	0.008353535
81	F	22	Chronic relapsing multiple osteomyelitis	Drug (−)	16.9	0.009005917	0.149704142	0.000209586	0.016976331
82	F	84	Sjögren disease	Drug (−)	17.1	0.005810526	0.193157895	0.000072983	0.006163743
83	F	54	Susp	Drug (−)	38.4	0.008794271	0.129036458	0.000105234	0.003119792
84	M	90	Susp	Drug (−)	31.8	0.008537736	0.136572327	0.000097988	0.004267296
85	F	33	Sjögren disease	Drug (+)	31.4	0.007398089	0.297547771	0.000061943	0.006439490
86	F	47	Susp	Drug (−)	15.2	0.006514474	0.200000000	0.000800000	0.009052632
87	M	49	Susp	Drug (−)	25.0	0.008432000	0.163240000	0.000135920	0.005620000
88	M	69	Kidney tumor	Drug (−)	27.2	0.009573529	0.155845588	0.000103971	0.006294118
89	M	17	Hives	Drug (−)	39.2	0.005017857	0.111581633	0.000360204	0.003076531
90	M	73	Susp	Drug (−)	19.3	0.013124352	0.125284974	0.000000000	0.004787047
91	F	79	Sjögren disease	Drug (−)	12.6	0.008333333	0.300079365	0.000315159	0.008396825
92	F	19	Sjögren disease	Drug (−)	20.2	0.013118812	0.274059406	0.000158020	0.004653465
93	F	53	Psoriatic arthritis	Drug (−)	30.0	0.009800000	0.130933333	0.000142367	0.005866667
94	F	62	Sjögren disease	Drug (−)	22.4	0.010625000	0.174151786	0.000000000	0.005834821
95	F	70	Sjögren disease	Drug (−)	18.6	0.004898925	0.217150538	0.000178280	0.006134409
96	F	69	Rheumatoid arthritis	Drug (+)	27.8	0.003488489	0.157877698	0.000178345	0.005665468
97	F	57	Osteoarthritis	Drug (−)	36.1	0.006637119	0.166648199	0.000000000	0.004288089
99	F	65	Sjögren disease	Drug (−)	31.0	0.008400000	0.198161290	0.000304710	0.005116129
100	F	65	Systemic lupus erythematosus	Drug (−)	12.9	0.029658915	0.459922481	0.000000000	0.030069767
101	M	41	Susp	Drug (−)	21.3	0.008497653	0.237511737	0.000000000	0.006995305
103	F	49	Rheumatoid arthritis	Drug (−)	17.8	0.012825843	0.271685393	0.000079719	0.006617978
104	F	25	Susp	Drug (−)	16.9	0.006088757	0.309881657	0.000199645	0.004502367
105	M	85	Remitting seronegative symmetrical synovitis with pitting edema	Drug (−)	9.8	0.006854082	0.328979592	0.000492041	0.017408163
106	M	64	Psoriatic arthritis	Drug (−)	31.4	0.007605096	0.163375796	0.000000000	0.002921975
108	F	39	Behçet’s disease	Drug (−)	31.9	0.005238245	0.222727273	0.000000000	0.009946708
109	F	69	Rheumatoid arthritis	Drug (+)	11.3	0.012566372	0.486548673	0.000000000	0.013752212
110	F	41	Behçet’s disease	Drug (+)	30.3	0.004590759	0.138943894	0.000110990	0.011861386
111	F	22	Adult onset Still’s disease	Drug (+)	8.6	0.01055814	0.820465116	0.000198953	0.020895349
112	F	69	Susp	Drug (−)	23.2	0.006672414	0.232887931	0.000000000	0.005293103
113	F	25	Autoinflammatory syndrome	Drug (−)	30.3	0.004541254	0.175874587	0.000000000	0.003871287
114	F	74	Sjögren disease	Drug (−)	52.8	0.008676136	0.116344697	0.000029299	0.002414773
115	M	77	Remitting seronegative symmetrical synovitis with pitting edema	Drug (−)	15.2	0.012328947	0.318157895	0.000152171	0.014559211
117	F	59	Susp	Drug (−)	30.7	0.009641694	0.193257329	0.000042541	0.003374593
118	F	78	Systemic Sclerosis	Drug (+)	12.4	0.012403226	0.261774194	0.000000000	0.013064516
119	F	76	Sjögren disease	Drug (+)	17.4	0.015873563	0.296839080	0.000170575	0.007614943
120	F	54	Susp	Drug (−)	41.8	0.016787081	0.173540670	0.000041794	0.003665072
121	F	73	Sjögren disease	Drug (−)	26.9	0.020750929	0.280148699	0.00024658	0.006234201
122	F	51	Osteoarthritis	Drug (−)	29.4	0.030843537	0.379251701	0.000127347	0.006261905
123	M	66	Rheumatoid arthritis	Drug (+)	12.6	0.014563492	0.358095238	0.000370952	0.012119048
124	F	47	Susp	Drug (−)	24.8	0.009866935	0.261814516	0.000122218	0.009302419
125	F	87	Susp	Drug (−)	27.2	0.010441176	0.207794118	0.000034533	0.005459559
126	F	63	Psoriatic arthritis	Drug (+)	22.7	0.008722467	0.225991189	0.000076960	0.005938326
127	F	54	Rheumatoid arthritis	Drug (−)	12.3	0.007803252	0.250569106	0.000144514	0.007589431
128	F	82	Osteoarthritis	Drug (−)	19.1	0.005732984	0.202827225	0.000033356	0.004887435
129	M	71	Rheumatoid arthritis	Drug (+)	30.5	0.016550820	0.181508197	0.000030269	0.003201967
130	F	67	Rheumatoid arthritis	Drug (−)	12.0	0.019025000	0.394750000	0.000044342	0.012375000
131	F	58	Dermatomyositis	Drug (−)	22.1	0.010656109	0.446877828	0.000022855	0.007104072
132	F	48	Rheumatoid arthritis	Drug (+)	13.0	0.027269231	0.442384615	0.000291000	0.010407692
133	M	75	Right knee monoarthritis	Drug (−)	23.0	0.009069565	0.181782609	0.000059174	0.007978261
134	F	53	Rheumatoid arthritis	Drug (−)	18.9	0.018629630	0.299047619	0.000348519	0.008661376
135	F	87	Systemic sclerosis	Drug (−)	46.8	0.003989316	0.112692308	0.000000000	0.003130342
136	M	24	Susp	Drug (−)	31.9	0.005442006	0.154796238	0.000000000	0.003247649
137	F	35	Inflammatory bowel disease	Drug (+)	20.0	0.005290000	0.148850000	0.000108250	0.007690000
138	F	57	Sjögren disease	Drug (+)	38.7	0.013227390	0.247338501	0.000040956	0.004198966
139	F	68	Rheumatoid arthritis	Drug (−)	25.9	0.012166023	0.272432432	0.000689961	0.005081081
141	M	20	Susp	Drug (+)	31.6	0.009110759	0.165727848	0.000089494	0.004952532
143	F	34	Susp	Drug (−)	31.2	0.009134615	0.231698718	0.000000000	0.005778846
144	F	50	Systemic lupus erythematosus	Drug (−)	26.0	0.019280769	0.211461538	0.000136231	0.005165385
145	F	21	Susp	Drug (−)	29.4	0.008037415	0.175068027	0.000085204	0.004489796
146	F	53	Sjögren disease	Drug (−)	31.9	0.006630094	0.187304075	0.000144514	0.004921630
147	F	43	Rheumatoid arthritis	Drug (−)	25.8	0.008313953	0.192713178	0.000238217	0.005116279
148	F	46	Susp	Drug (−)	24.1	0.014506224	0.349377593	0.000000000	0.006468880
150	F	56	Susp	Drug (−)	41.1	0.013165450	0.161849148	0.000186034	0.004654501
151	F	59	Susp	Drug (−)	20.4	0.013446078	0.211421569	0.000811765	0.007181373
152	F	55	Systemic sclerosis	Drug (−)	32.8	0.020307927	0.276097561	0.000124482	0.005006098
153	M	72	Susp	Drug (−)	32.5	0.015803077	0.134123077	0.000000000	0.003624615
154	F	77	Systemic sclerosis	Drug (−)	30.5	0.015767213	0.191180328	0.000055115	0.005632787
155	F	16	Behçet’s disease	Drug (+)	40.1	0.007750623	0.126159601	0.000057880	0.003743142
156	F	76	Susp	Drug (−)	33.6	0.015127976	0.172946429	0.000243036	0.003041667
157	F	48	Seronegative RAr/o	Drug (−)	24.7	0.018040486	0.257449393	0.000183401	0.004801619
159	F	89	Rheumatoid arthritis	Drug (+)	39.1	0.007544757	0.098388747	0.000079130	0.002879795
160	F	83	Rheumatoid arthritis	Drug (+)	16.9	0.008059172	0.162662722	0.000395207	0.005758580
161	F	87	Rheumatoid arthritis	Drug (+)	38.4	0.014841146	0.110390625	0.000049271	0.004505208
162	F	82	Rheumatoid arthritis	Drug (+)	54.5	0.005761468	0.082220183	0.000050642	0.002016514
163	F	45	Sjögren disease	Drug (−)	36.0	0.007208333	0.168861111	0.000218361	0.003916667
164	F	61	Rheumatoid arthritis	Drug (+)	15.6	0.007923077	0.359807692	0.000000000	0.009294872
165	F	56	Rheumatoid arthritis	Drug (+)	32.1	0.009448598	0.151183801	0.000092804	0.002928349
166	F	74	Rheumatoid arthritis	Drug (+)	38.3	0.011595300	0.160939948	0.000000000	0.003086162
167	F	66	Rheumatoid arthritis	Drug (+)	24.0	0.012333333	0.255041667	0.000051458	0.006679167
168	F	43	Rheumatoid arthritis	Drug (+)	27.1	0.013730627	0.127490775	0.000000000	0.004011070

**Table 3:** ITGα7 and LAMα4 expression in outpatients with Sjögren disease at the Division of Diabetes, Endocrinology and Clinical Immunology, Department of Internal Medicine, Hyogo Medical University.

Block Number	Age	Greenspan	ITGα7	LAMα4
118-07958	68	1	17.879741	8.525875
118-08270	41	1	3.118290	1.736562
118-09379	63	1	11.370192	7.534245
119-07606	50	1	0.544213	5.782326
118-10710	42	2	5.152732	2.928132
118-12450	42	2	10.895517	10.400495
118-06150	77	3	15.515302	24.815080
118-12177	69	4	11.029780	17.175083
119-12626	81	4	7.521222	11.195179
119-12891	69	4	4.017859	9.960443

## Data Availability

The original contributions presented in this study are included in the article/Supplementary Materials. Further inquiries can be directed to the corresponding author.

## Supplementary Materials

The following supporting information can be downloaded at: https://www.mdpi.com/article/10.3390/ijms27167307/s1.

## References

[1] Taniguchi K., Yoshikawa T., Yanagita M. (2025). The roles of tertiary lymphoid structures in orchestrating immune responses in peripheral organs. Inflamm. Regen..

[2] Fridman W.H., Meylan M., Pupier G., Calvez A., Hernandez I., Sautès-Fridman C. (2023). Tertiary lymphoid structures and B cells: An intratumoral immunity cycle. Immunity.

[3] Hu C., You W., Kong D., Huang Y., Lu J., Zhao M., Jin Y., Peng R., Hua D., Kuang D.M. (2024). Tertiary Lymphoid Structure-Associated B Cells Enhance CXCL13+CD103+CD8+ Tissue-Resident Memory T-Cell Response to Programmed Cell Death Protein 1 Blockade in Cancer Immunotherapy. Gastroenterology.

[4] Suzuki N., Nishiura H., Okuno M., Imasaka M., Sugimoto M., Nakamura I., Tachibana T., Fujimoto J., Ohmuraya M. (2026). Association Between CD4+/Programmed Cell Death 1+/CD153+ Cell Infiltration to the Aging Liver and Progression of Hepatic Steatosis in a Novel Metabolic Dysfunction-Associated Steatohepatitis Model Nishiura Mouse. Lab. Investig..

[5] Nishiura H., Matsui K., Azuma N., Hashimoto T., Chen W., Yamanegi K., Ohe C., Sugimoto M., Imasaka M., Ohmuraya M. (2026). Senescence-Associated Tertiary Lymphoid Structures in Sjögren’s Disease Model Nishiura Mice. Mech. Ageing Dev..

[6] Sugiyama T., Omatsu Y., Nagasawa T. (2019). Niches for hematopoietic stem cells and immune cell progenitors. Int. Immunol..

[7] Morikawa T., Takubo K. (2016). Hypoxia regulates the hematopoietic stem cell niche. Pflug. Arch..

[8] Takubo K., Suda T. (2012). Roles of the hypoxia response system in hematopoietic and leukemic stem cells. Int. J. Hematol..

[9] Su Y., Iacob R.E., Li J., Engen J.R., Springer T.A. (2022). Dynamics of integrin α5β1, fibronectin, and their complex reveal sites of interaction and conformational change. J. Biol. Chem..

[10] Chigaev A., Zwartz G., Graves S.W., Dwyer D.C., Tsuji H., Foutz T.D., Edwards B.S., Prossnitz E.R., Larson R.S., Sklar L.A. (2003). Alpha4beta1 integrin affinity changes govern cell adhesion. J. Biol. Chem..

[11] Soldi R., Mitola S., Strasly M., Defilippi P., Tarone G., Bussolino F. (1999). Role of alphavbeta3 integrin in the activation of vascular endothelial growth factor receptor-2. EMBO J..

[12] Lassiter C.M., Gal J.S., Becker S., Hartman N.W., Grabel L. (2016). Embryonic stem cell-derived neural progenitors transplanted to the hippocampus migrate on host vasculature. Stem Cell Res..

[13] Zhang D., Yang S., Toledo E.M., Gyllborg D., Saltó C., Carlos Villaescusa J., Arenas E. (2017). Niche-derived laminin-511 promotes midbrain dopaminergic neuron survival and differentiation through YAP. Sci. Signal..

[14] Machida H., Yokota J., Fujiwara H. (2026). Basement membrane components define the microenvironment of aggregated fibroblasts in the skin and support their aggregation in vitro. Matrix Biol..

[15] Yoshiba N., Maekawa T., Sekiguchi K., Kaku M., Sirisereephap K., Surboyo M., Sato-Yamada Y., Rosenkranz A., Hosoya A., Ohkura N. (2025). Loss of integrin alpha7-mediated signaling induces a dendritic cell-like phenotype in macrophages cultured on laminin-211/221 isoforms. J. Biol. Chem..

[16] Schöber S., Mielenz D., Echtermeyer F., Hapke S., Pöschl E., von der Mark H., Moch H., von der Mark K. (2000). The role of extracellular and cytoplasmic splice domains of alpha7-integrin in cell adhesion and migration on laminins. Exp. Cell Res..

[17] Saher G., Hildt E. (1999). Activation of c-Raf-1 kinase signal transduction pathway in alpha(7) integrin-deficient mice. J. Biol. Chem..

[18] Sato Y., Oguchi A., Fukushima Y., Masuda K., Toriu N., Taniguchi K., Yoshikawa T., Cui X., Kondo M., Hosoi T. (2022). CD153/CD30 signaling promotes age-dependent tertiary lymphoid tissue expansion and kidney injury. J. Clin. Investig..

[19] Xie G., Chen X., Gao Y., Yang M., Zhou S., Lu L., Wu H., Lu Q. (2025). Age-Associated B Cells in Autoimmune Diseases: Pathogenesis and Clinical Implications. Clin. Rev. Allergy Immunol..

[20] Takano T., Kotaki R., Park J., Yoshida T., Wakatsuki Y., Tanokura M., Miyakawa T., Takahashi K., Nakajima-Adachi H., Hachimura S. (2020). Age-Dependent Decrease in the Induction of Regulatory T Cells Is Associated with Decreased Expression of RALDH2 in Mesenteric Lymph Node Dendritic Cells. Front. Immunol..

[21] Li L., Shirkey M.W., Zhang T., Piao W., Li X., Zhao J., Mei Z., Guo Y., Saxena V., Kensiski A. (2022). Lymph node fibroblastic reticular cells preserve a tolerogenic niche in allograft transplantation through laminin α4. J. Clin. Investig..

[22] Sobel R.A., Hinojoza J.R., Maeda A., Chen M. (1998). Endothelial cell integrin laminin receptor expression in multiple sclerosis lesions. Am. J. Pathol..

[23] Kutlesa S., Siler U., Speiser A., Wessels J.T., Virtanen I., Rousselle P., Sorokin L.M., Müller C.A., Klein G. (2002). Developmentally regulated interactions of human thymocytes with different laminin isoforms. Immunology.

[24] Naito Y., Hino K., Bono H., Ui-Tei K. (2015). CRISPRdirect: Software for designing CRISPR/Cas guide RNA with reduced off-target sites. Bioinformatics.

