# Supplementary material for: Integrin α7-Driven Senescence-Associated Follicular Helper T Cell Adhesion Induces Tertiary Lymphoid Structures in Nishiura Mice
Source: Int J Mol Sci. 2026 Aug 16;27(16):7307. doi: 10.3390/ijms27167307 (PMC13513366; doi:10.3390/ijms27167307)
Supplement: Supplementary file 1 [file ijms-27-07307-s001.zip › ijms-4478178-supplementary file-conversion.pdf]

Supplementary material

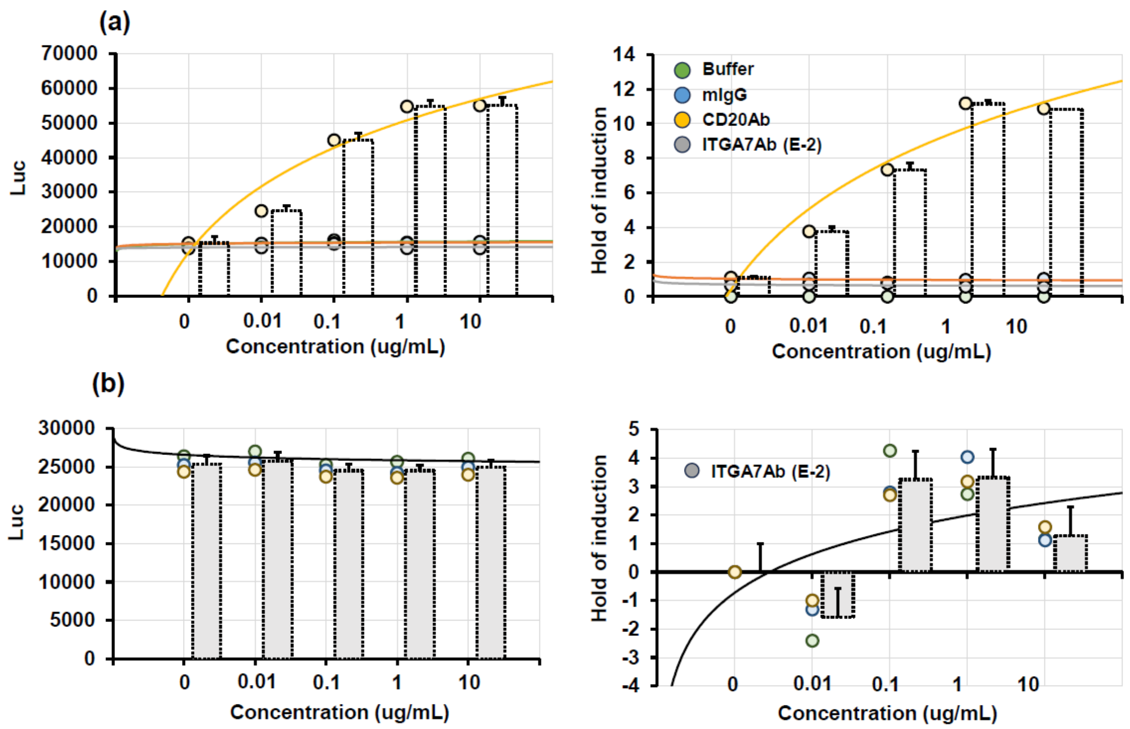

Figure S1. Antibody-Dependent Cellular Cytotoxicity Bioassay. (a) Antibody-Dependent Cellular Cytotoxicity (ADCC) bioassay target cells (hRaji) or (b) mSA-Tfh cells (1×10^{4}) were seeded into 96-well flat-bottom white plates using the ADCC Reporter Bioassay Complete Kit (hRaji). Ten μL of mIgG, anti-CD20 antibody, or anti-ITG*α*7 mIgG (E-2) was added to achieve final concentrations of 0–10 μg/mL, followed by incubation at 37°C in 5% CO_2. After 30 minutes, six-fold excess ADCC effector cells (CD20-expressing B cell-derived cells) were added. Following 6 hours of incubation, 60 μL Bio-Glo™ Luciferase Assay Reagent was added and incubated for 30 minutes. ADCC activity was quantified as luciferase activity using a GloMax 96 luminometer.

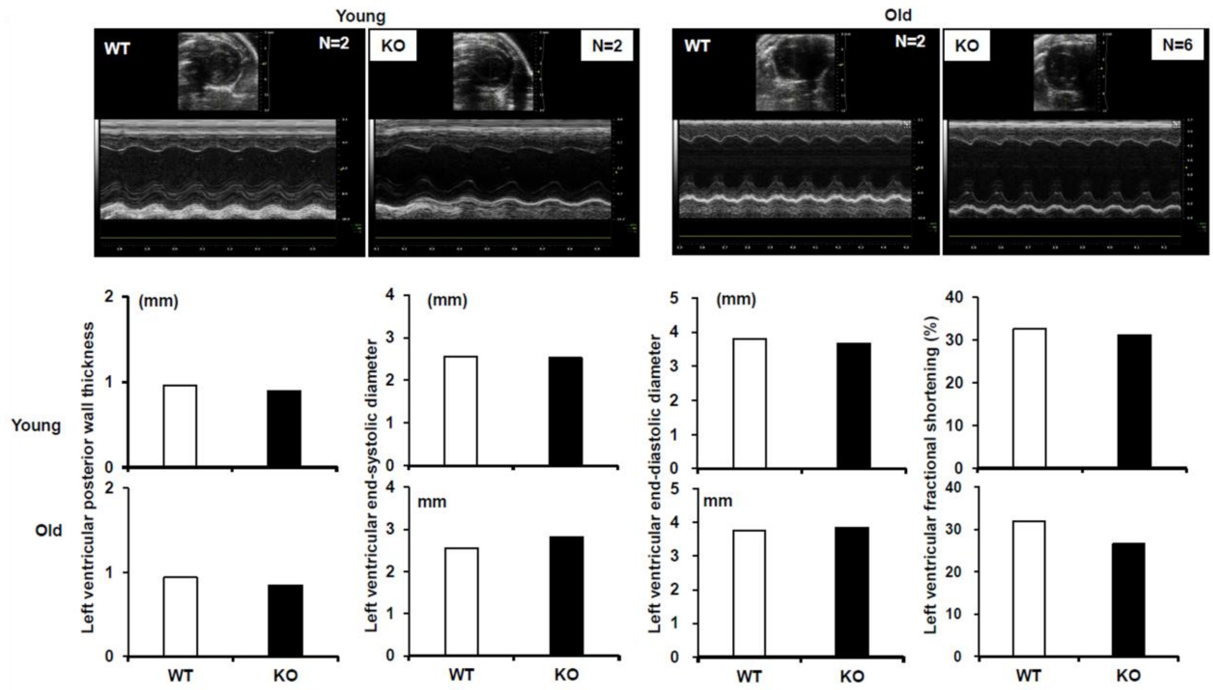

Figure S2. Side Effects of ITGα7 Deficiency. Twelve-week-old (young) and 42-week-old (aged) C57BL/6 (B6) female mice (N = 2 per age group) and ITGα7 KO mice (N = 2 per age group) were anesthetized with 2% isoflurane and placed on a heated platform. Cardiac function, including left ventricular end-diastolic diameter, end-systolic diameter, and fractional shortening was assessed using a high-frequency ultrasound system. Two-dimensional parasternal short-axis images were obtained at the level of the papillary muscles, and M-mode tracings were recorded for quantitative analysis.

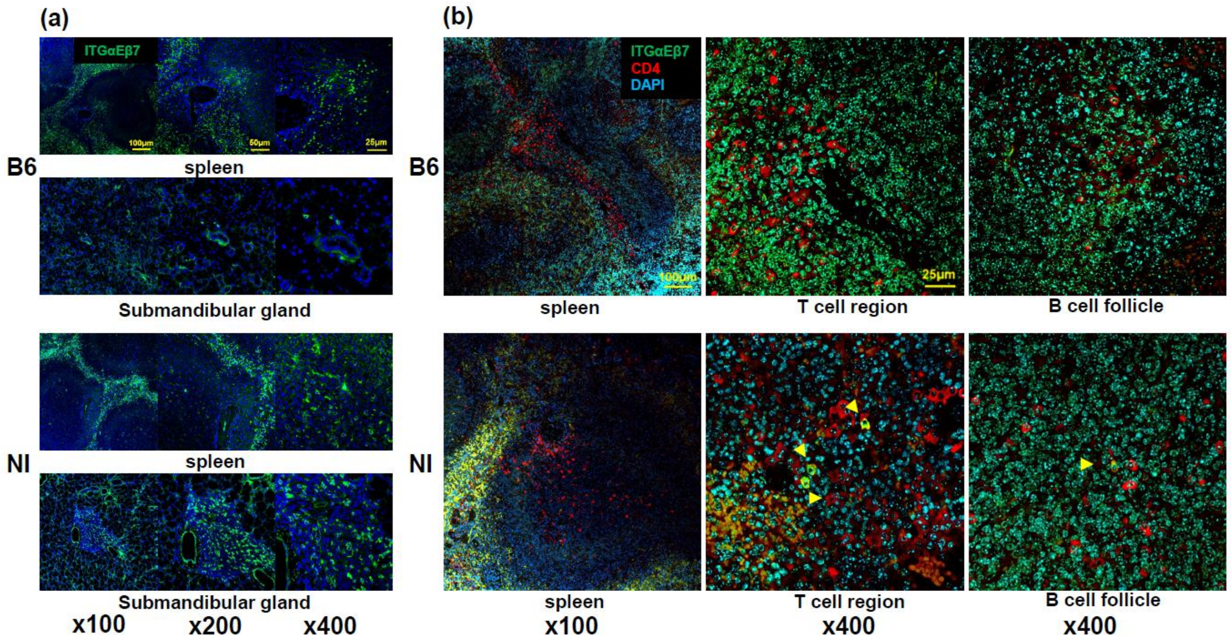

Figure S3. Expression of ITGαEβ7 in Nishiura Mice. (a) Paraffin sections of spleens and submandibular glands from C57BL/6 (B6) and Nishiura (NI) mice were incubated with anti-CD103 (ITGαEβ7) rabbit IgG at 4°C for 24 hours. Sections were then incubated at 22°C for 2 hours with Alexa Fluor™ 488-conjugated anti-mouse IgG (H+L) and DAPI. (b) Adjacent paraffin sections were incubated at 4°C for 24 hours with anti-ITGαEβ7 rabbit IgG together with anti-CD4 mouse IgG. Sections were then incubated at 22°C for 2 hours with Alexa Fluor™ 488-conjugated anti-rabbit IgG and Alexa Fluor™ 647-conjugated anti-mouse IgG, along with DAPI. RGB images were acquired using a Zeiss LSM780 confocal laser scanning microscope.
